# Miscibility of poly(acrylic acid)/poly(methyl vinyl ketone) blend and in vitro application as drug carrier system

**DOI:** 10.1080/15685551.2018.1521563

**Published:** 2018-09-22

**Authors:** Abdulaziz Ali Alghamdi, Abdulellah Alsolami, Waseem Sharaf Saeed, Abdel-Basit Mohammed Al-Odayni, Abdelhabib Semlali, Taieb Aouak

**Affiliations:** a Chemistry Department, College of Science, King Saud University, Riyadh, Saudi Arabia; b Biochemistry department, College of Science, King Saud University, Riyadh, Saudi Arabia

**Keywords:** Poly(acrylic acid)/poly(methyl vinyl ketone) blend, sulfamethoxazole, in vitro drug release, mass transfer

## Abstract

A series of poly(acrylic acid)/poly(methyl vinyl ketone) (PAA/PMVK) blends with different compositions were prepared by the solvent casting method. The miscibility of this pair of polymers was investigated by differential scanning calorimetry(DSC), Fourier transform infra-red (FTIR) and X-Ray diffraction (XRD) techniques. An *in-vitro* cytotoxicity test of the drug-carrier system via MTT (3-(4,5-demethylthiazol-2-yl)-2,5-diphenyltetrazolium bromide) assay revealed no significant cytotoxic effects at concentrations up to 100 µg· ml^−1^. The STX/PAA-50 drug carrier systems were also prepared by solvent casting of solutions containing the sulfamethoxazole (STX) used as drug model and PAA/PMVK blend in *N.N*-dimethylformamide then crosslinked with acidified ethylene glycol. The release dynamic of STX from the prepared hydrogels was investigated in which the diffusion through the polymer matrix, the enhancement of the water solubility of STX, the influence of the initial drug concentration, the pH of the medium, and the effect of the degree of swelling of the polymer matrix on the release dynamic was evaluated. According to the total gastrointestinal transit time estimated by Belzer, the estimate distribution of STX released in the different organs indicated that the performance is obtained with the drug – carrier-system containing equal ratios of polymer and 10 wt% of STX (STX-10/PAA-50).

## 1. Introduction

The use of synthesized polymers in the biomedical domain gained considerable attention from different researchers in the last decade [1–8]. A wide range of these polymers are available, and have further advantages to be tunable in biological, physic-chemical, mechanical and optical properties to match the requirements of specific applications. However, many of them need modification in order to acquire supplementary desirable properties which are not found in one polymer. Many featured Polymeric materials can be prepared by copolymerization of two or more different monomers having complementary desirable properties. The main disadvantages of this route are the complexity in its realization, costly and difficulty on physical properties control by compositional changes [9–11]. Blending polymers offer a key option in solving emerging application requirements. The ability to combine existing polymers into new compositions with marketable properties offers the advantages of reduced research, cost and development expense compared to the development of new monomers and polymers to yield a similar property profile. The development of so-called ‘Smart polymers’ is anticipated to lead to targeted delivery systems, in which a particular formulation can be directed to specifically target cells, tissues, or other desired sites. Controlled drug carrier systems generally comprise a polymer, whether natural or synthetic, that is judiciously combined with a drug in such a way that the drug is released from this material in a predetermined manner. The release of the drug must be stable over a long period and maintains the drug level within a desired range. The ideal drug carrier system should be non-toxic, biocompatible, biodegradable at some point, inert, mechanically strong, comfortable for the patient, facilitate high drug loading, safe from accidental release, simple to administer and remove, and easy to fabricate and sterilize [12,13]. Due to their desirable properties, many polymeric materials that originally intended for non-biological uses were applied to the controlled release of drugs afterward [14–16]. Poly(acrylic acid) (PAA) is an anionic polyelectrolyte that can be easily synthesized via free radical polymerization. The hydrogel of PAA, which is obtained by a crosslinking reaction using chemical or physical methods has the capacity to swell two hundreds of times of its initial weight [17]. PAA is a water soluble, biodegradable [18–24] and pharmaceutically safe polymer that considerably employed in a wide range of medicinal applications [19]. According to Calixto [25], PAA produces a hydrogel that is promising for topical drug delivery, allowing close contact with a biological substrate as well as enhancement of the local concentration gradient, where both factors may improve the biological performance of the drugs. In the last decade, various researchers have focused on developing new drug-carrier systems comprising nanogels based on PAA materials [26–28]. Müller et al. [29] investigated the oral drug delivery application of mucus-penetrating papain combined with a PAA nanoparticle composite (PAPC). This system showed remarkable ability to cleave the mucogly-*co*-protein substructures responsible for the structural and rheological properties of mucus. Moreover, the permeation behavior of PAPC particles was remarkably enhanced as result of local mucus disruption by papain. Recently, a nanocomposite PAA nanogel combined with biodegradable poly(hydroxybutyrate) (PHB) was employed in bone regeneration and drug delivery applications by Larsson and coworkers [30]. As a result, these nanocomposites were prospectively useful as dual-functional scaffolds for bone regeneration. A dextran-poly(acrylic acid) copolymer as a drug carrier of ibuprofenwas investigated by Guo et al. [31]. The study revealed that the substituent carboxyl groups of poly(acrylic acid) interact strongly with the proton acceptors of the glucose units in dextran. This copolymer showed a smart pH response, where they shrank in acidic media and swelled in high-pH media. The pH-dependent response of acrylic acid-based polymers were also investigated as controlled drug carrier systems. A polymeric matrix comprising Linseed (Linum usitatissimum), L. hydrogels (LSH) was prepared by free radical copolymerization rout between acrylic and methacrylic (MAA) acids using *N, N*-methylene bis-acrylamide (MBA) as a crosslinking agent, and investigated as drug carrier of ketoprofen by Shabir et al. [32] It was found that swelling of the copolymer hydrogels was the greatest at pH 7.4. The extent of ketoprofen release was found to increase as the monomer content increased, and decreasewhen the MBA content decreased. Furthermore, the dynamic release followed Korsmeyer-Peppas model.

Poly(methyl vinyl ketone) (PMVK) is practically unknown in the biomedical field due to its insolubility in water, among other unfavorable properties. This polymer is generally synthesized via free radical polymerization of methyl vinyl ketone (MVK). The presence of the carbonyl group in the substituent of this polymer provides hydrogen bond with polymer having hydroxyl or carboxyl acid group such as PAA favoring their miscibility.

Sulfamethoxazole (STX) is a medication used alone or in combination with trimethoprim to treat a large variety of bacterial infections and certain types of pneumonia, such as pneumocystis [33]. However, this medication also causes side effects such as gastrointestinal disturbances, linked to nausea, vomiting, and anorexia [34,35]. Chemically, it can react easily with many compounds through its active amine group, especially with those containing ketone groups, through the Schiff base reaction. Such modifications may reduce some of the undesirable effects [36,37]. In order to control the hydrophilicity of PAA hydrogel to obtain competitive material having performing properties in drug carrier domain we have prepared a series of PAA/PAMVK blends with different compositions by solvent casting method. The obtained miscibility of this pair of polymer was characterized by differential scanning calorimetry (DSC) and Fourier transform infrared (FTIR). The mass transfer of water through the PAA/PMVK hydrogels in which the Fick model was verified was also studied. STX/PAA/PMVK drug-carrier systems were prepared by solvent casting process in which the homogenous polymer mixture was mixed with STX at different ratios. The obtained systems were characterized by DSC, XRD, SEM methods. The MTT assay was applied to assess the in vitro cytotoxicity of the drug carrier systems, and the cell adhesion and cell growth on the PAA/PMVK hydrogel. The dynamics of STX release was investigated in depth by analyzing the diffusion through the polymer matrix, the influence of the initial drug loading, the pH of the medium, and the swelling behavior of the hydrogel. Moreover, water solubility enhancement of SMX was also evaluated.

## 2. Experimental

### 2.1. Materials

AA (purity 99%, Sigma Aldrich) is purified from the hydroquinone (inhibitor of radical polymerization) by distillation under reduced pressure prior to use. PMVK (M_n_ = 5 x 10^5^ g.mol^−1^, Sigma Aldrich). 2,2-Azo-bis-isobutyronitrile (AIBN, 98%, Aldrich) is purified twice by recrystallization in ethanol. 4-amino-*N*-(5-methylisoxazol-3-yl)-benzenesulfonamid (SMX) is extracted from Septrin tablets manufactured by Glaxo, Saudi Arabia, using the reported method [38]. Ethylene glycol (EG, 99.8%, Sigma), tetrahydrofuran (THF, 99.5%, Alpha Chimica), and *N, N*-dimethyl formamide (DMF, 99.8%, Scharlau Company) are used without further purification.

### 2.2. Preparation of PAA/PMVK blend

**Scheme 1.**
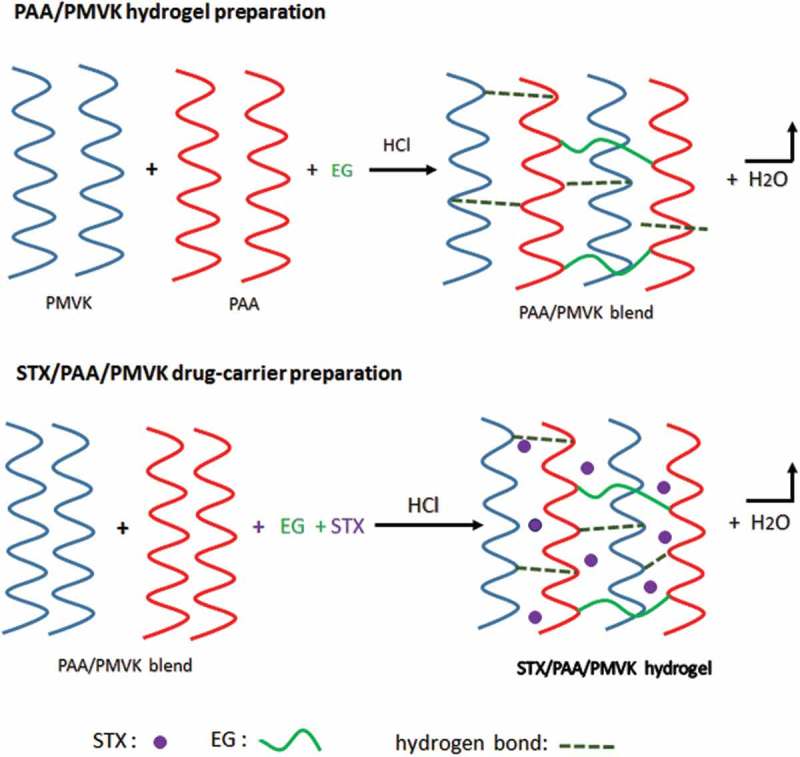
Schematization of PAA/PMVK hydrogel formation (up) and formation of STX/PAA/PMVK drug carrier (below).

PAA is synthesized in THF from AA via free radical polymerization. 0.12 mmol (2 mg) of AIBN is introduced in a two necked flask containing a magnetic stirrer bar maintained in nitrogen gas atmosphere then 0.2 mol (14.4 g) of AA amount is added to the reactor. The polymerization temperature is fixed at 60°C during all the reaction process until observing a very viscous solution indicating the polymer formation. The polymer obtained is then precipitated in *n*-heptane and dried for 24 h at room temperature and then under vacuum at 60°C for 12 h. A series of PAA/PMVK blends containing 10, 25, 50, 75 and 90 wt% of PAA are prepared by dissolution in THF under continuous stirring and their formulas are grouped in Table 1. PAA/PMVK hydrogel which is used in the swelling test, is prepared by dissolution of the 6.0 g of the corresponding blend and 0.001 g of EG in 40 mL of THF acidified with 0.5 mL of 0.1 N hydrochloric acid (Scheme 1, in up). All PAA/PMVK films are prepared by smoothly casting the polymeric solutions over a perfect horizontal Teflon-plate surface obtained using a spirit level. To remove the residual EG used as crosslinking agent in the PAA/PMVK hydrogel, the produced film was washed several times with methanol and dried again as before to constant weight.

**Table 1.** Preparation conditions of PAA/PMVK blend with different compositions.

System	PAA (g)	PMVK(g)	THF(mL)
PAA-10	1.0	9.0	25
PAA-25	2.5	7.5	25
PAA-50	5.0	5.0	25
PAA-75	7.5	2.5	25
PAA-90	9.0	1.0	25

### 2.3. Preparation of STX/PAA/PMVK drug-carrier system

In a 100-mL flask, a certain amount of PAA/PMVK blend containing 40 wt% of PMVK is completely dissolved in 50 mL of THF at 25 °C under continuous stirring to prepare polymeric solution-A. In another 100 mL-flask, a certain amount of STX and ethylene glycol acidified with 0.5 mL of 0.1 N hydrochloric acid were dissolved in 10 mL of THF with stirring until complete dissolution, forming solution-B. These two solutions are mixed to form a new solution composed of two polymers, drug, crosslinking agent and solvent (Scheme 1, in below). A series of STX/PAA/PMVK films containing equal amount of polymer components and 3 (STX-3/PAA50), 5 (STX-5/PAA50), 7 (STX-7/PAA50) and 10 wt% (STX-10/PAA50) of STX contents are prepared by this same method and the preparation conditions are gathered in Table 2. The thickness of each film measured by a digital micrometer is ranged between 127 and 264 µm.

**Table 2.** Preparation conditions of STX/PAA-50 systems.

System	STX(wt%)	PAA(g)	PMVK(g)	Polymer(wt%)
STX-3/PAA-50	3	48.5	48.5	97
STX-5/PAA-50	5	47.5	47.5	95
STX-7/PAA-50	7	46.5	46.5	93
STX-10/PAA-50	10	45.0	45.0	90

### 2.4. Characterization

The DSC thermograms of PAA/PMVK blend and STX/PAA/PMVK composites and their components are obtained using Shimadzu DSC 60A which previously calibrated with indium. Samples weighing between 8 and 10 mg are packed in aluminum DSC pans before placing in the DSC cell. The samples are scanned from −100 to + 200°C under nitrogen atmosphere at a heating rate of 20°C.min^−1^. The obtained thermograms reveals that blend, composites and their components does not undergo degradation. Glass transition temperatures, *T_g_*, are derived accurately from the thermograms as the midpoint in the heat capacity variation with temperature. To eliminate all eventual volatile compounds incrusted in the polymer, such as the solvent and residual monomer, the *T_g_* values are taken from the second run of the DSC process. The FTIR spectra of the PAA/PMVK blends and their components are obtained at 25 °C using a Perkin Elmer 1000 spectrophotometer. In all the cases, at least 32 scans, with an accuracy of 2 cm^−1^, were signal-averaged. The film samples were sufficiently thin and transparent to obey the Beer–Lambert law. The crystalline structure of the pure STX and that incorporated in the PAA/PMVK blend are examined by XRD analysis using an X-ray diffractometer (Rigaku Dmax 2000). The samples are directly used as films and analyzed using a Cu anode tube, tube voltage of 40 KV/40 mA, and generator current of 100 mA. The samples are then scanned in the 2θ range of 5–60° at a scanning rate of 1.0 °.min^−1^. The STX amounts released from the drug-carrier systems are determined by UV-visible using an Evolution 600 UV-Visible Spectrophotometer (Thermo Scientific) at 264 nm corresponding to the maximum absorption of this drug in water.

The effect of the PAA/PMVK and STX/PAA-50 hydrogels on Lovo and HCT-116 cell toxicity is determined by the MTT colorimetric assay described by Semlali and coworkers [39,40]. In brief, 3 × 10^5^ cells (Lovo and HCT-116)/well are seeded into 6-well plates and exposed to different concentrations of PAA/PMVK and STX/PAA-50 systems for 24 h. At the end of exposure, each culture medium are replaced with new medium containing MTT solution (5mg· ml^−1^ in PBS) in an amount equal to 10% of the culture volume, and incubated for 3 h at 37°C. The resulting formazan product is dissolved in 1.0 ml of 0.1% HCl-isopropanol solution. A 200 µl aliquot of the supernatant is transferred to a 96-well plate and the absorbance is measured at 550 nm using an X Mark Microplate Spectrophotometer (Bio-Rad, USA).

The surfaces and the cross-section morphologies of the PAA/PMVK blend and STX/PAA-50 drug-carrier system before and after the release process are examined by a JEOL JSM 6360 scanning electronic microscope using an acceleration voltage of 15 kV. The specimens are carefully coated with a thin layer of gold to reduce any buildup deposed on the film surfaces. The coating is realized using a JEOL JFC-1600 Auto Fine Coater operated at 20 mA for 80 s prior to the SEM analysis. The swelling measurements is performed on known dried PAA/PMVK hydrogels immersed into water at 37°C in different media pHs until no change in mass is observed.

## 3. Results and discussions

### 3.1. Miscibility

#### 3.1.1. Preliminary test

The observation of only one phase in each PAA/PMVK solution in THF and a transparent film obtained after solvent evaporation is an indication of the miscibility of this pair of polymers. The same remark is also observed for the STX/PAA-50 solutions.

#### 3.1.2. DSC analysis

**Figure 1.**
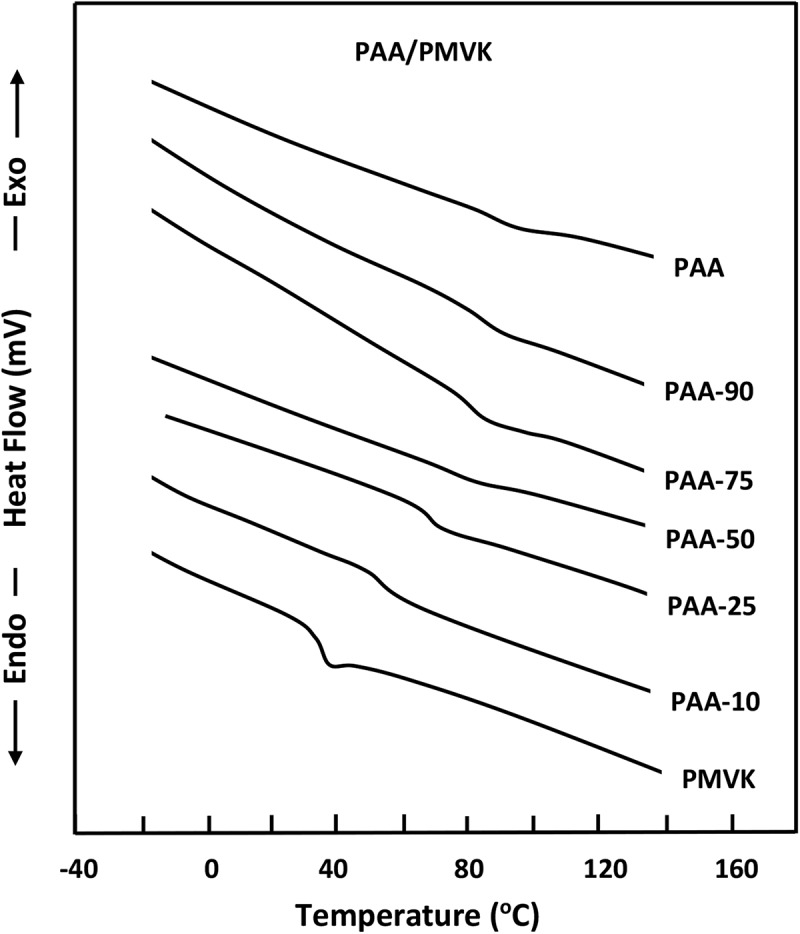
DSC thermograms of PAA/PMVK blend with different compositions performed with a heating rate of 20 °C.min^−1.^

The DSC thermograms for blends and their pure components are presented in Figure 1 and reveal glass transition temperatures, *T_g_*, at 90 °C and 35°C for pure PAA and pure PMVK, respectively, which perfectly agree with those of the literature [41,42]. However, those of PAA/PMVK blends show only a single *T_g_* for each composition localized between those of its components which decreased with increasing the PMVK content. According to Qui et al [43], the appearance of a single *T_g_* for a blend indicates a full miscibility in 20–40 nm scale.

**Figure 2.**
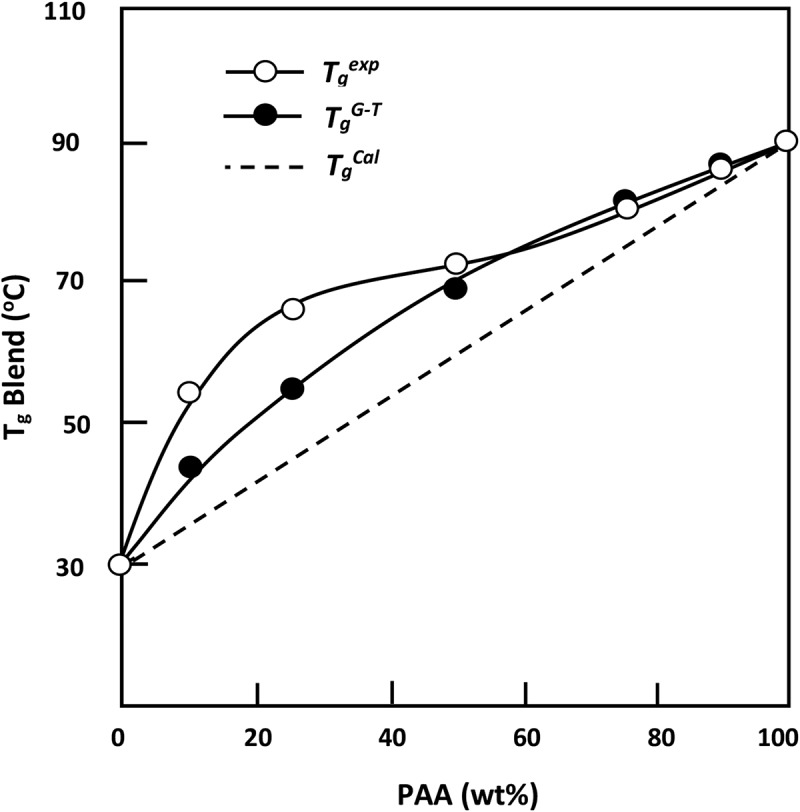
Variations of the *T_g_^exp^, T_g_^Cal^, T_g_^Fox^* and *T_g_^G-T^* of PAA/PMVK blend versus the composition.

The miscibility of PAA/PMVK system is also confirmed through the comparison of the curve profile indicating the variation of the experimental *T_g_* value versus the composition of the blend with those calculated from the Fox (Equation 1) [44] and Gordon Taylor (Equation 2) [45] using the experimental *T_g_s* of the pure components.

(1) $$\frac{1}{T_{gblend}}=\frac{w_{1}}{{T_{g}}_{1}}+\frac{w_{2}}{{T_{g}}_{2}}$$

(2) $${T_{g}}_{Blend}=\frac{w_{1}{T_{g}}_{1}+k(1-w_{1})Tg_{2}}{w_{1}+k(1-w_{1})}\;with\;k=\frac{\Delta \alpha_{1}}{\Delta \alpha_{2}}$$

(3) $$f_{free}^{CO}=\frac{A_{free}^{CO}}{A_{free}^{CO}+\;aA_{Asso}^{CO}}$$

where *w_i_* and *T_gi_* are the weight fraction and the glass transition temperature of polymer(*i*). *k* is an adjustable fitting parameter in the Gordon–Taylor equation, and *∆α_i_* is the change in the expansion coefficient at *T_gi_*. As can be seen from the obtained results plotted in Figure 2, the *T_g_* values of the blends fit reasonably well with those calculated using both the Fox and Gordon–Taylor equations with *k *= 0.4 and slightly deviate from those calculated from the arithmetic mean, *T_g_^calc^*. As such behavior indicates the miscibility of the components in all PAA/PMVK ratios.

**Figure 3.**
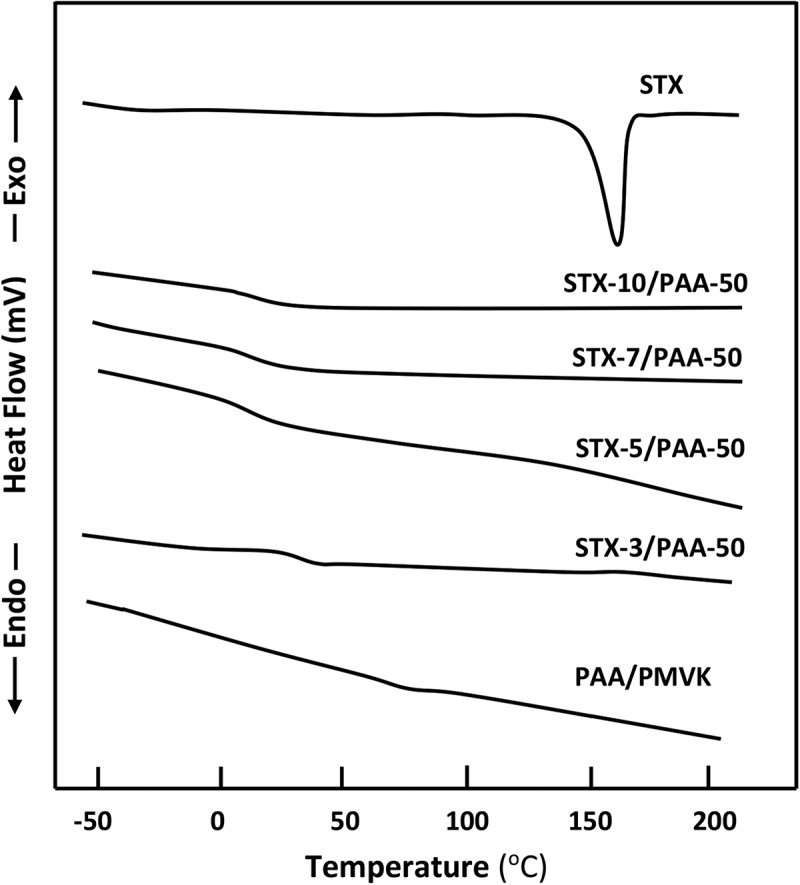
DSC thermograms of STX/PAA-50 blend with different compositions performed with a heating rate of 20 °C.min^−1.^

The DSC thermograms of STX/PAA-50 system, containing different STX contents are gathered in Figure 3 and show only one glass transition temperature for each composition and shifted toward the lower temperatures when the STX content decreased. This finding indicates that the addition of a small amount of STX to the binary PAA-50 system does not affect its miscibility. However, the comparison between the thermograms of PAA-50 blend with those of STX/PAA-50 drug-carrier systems also reveals a shift in this *T_g_* values toward the low temperatures. This phenomenon can be explained by an increase of a chain sliding due to the insertion of the STX particles between the polymer chains in the blend thus playing the role of plasticizer.

#### 3.1.3. FTIR analysis

**Figure 4.**
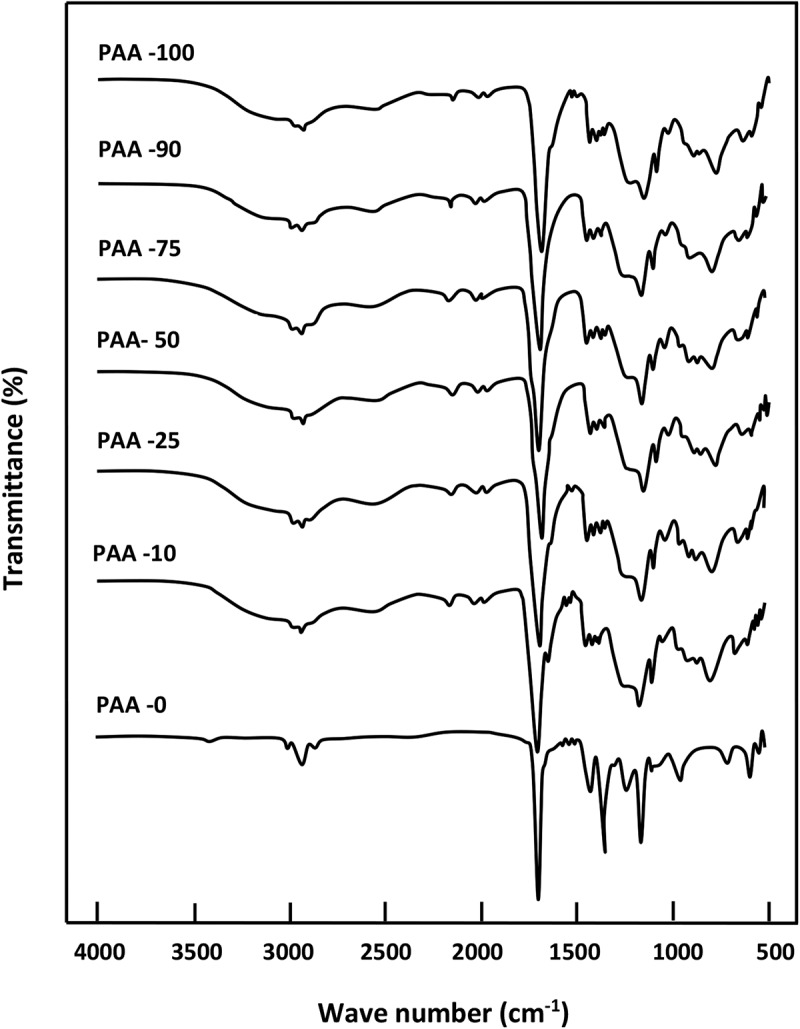
FTIR spectra of pure PAA, pure PMVK and PAA/PMVK blend with different compositions.

The presence of chemical groups in a polymer blend often leads to the creation of different interactions. Thereby, FTIR spectroscopy is commonly used to explore these types of interactions [46,47]. Indeed, it is particularly suitable for the detection of specific interactions created from the formation of hydrogen bonds. However, it is well known that the interactions resulting from the hydrogen bonds formation occurs in any blend containing a proton donor and a proton acceptor groups. The strength of the bond considerably affects the energy of the covalent bonds on the interactive species, therefore, a shift in the absorption band can be observed in the FTIR spectrum. Indeed, the stretching frequency of the acceptor group, such as the carbonyl group, is also shifted to the lower absorption bands (longer wavelengths), usually with an increase of the intensity of the hydrogen bond. Indeed, in the carbonyl region, Figure 4 shows the PAA spectrum which is dominated by the characteristic band of self-association attributed to the intermolecular carboxylic acid dimer at 1713 cm^–1^.

**Figure 5.**
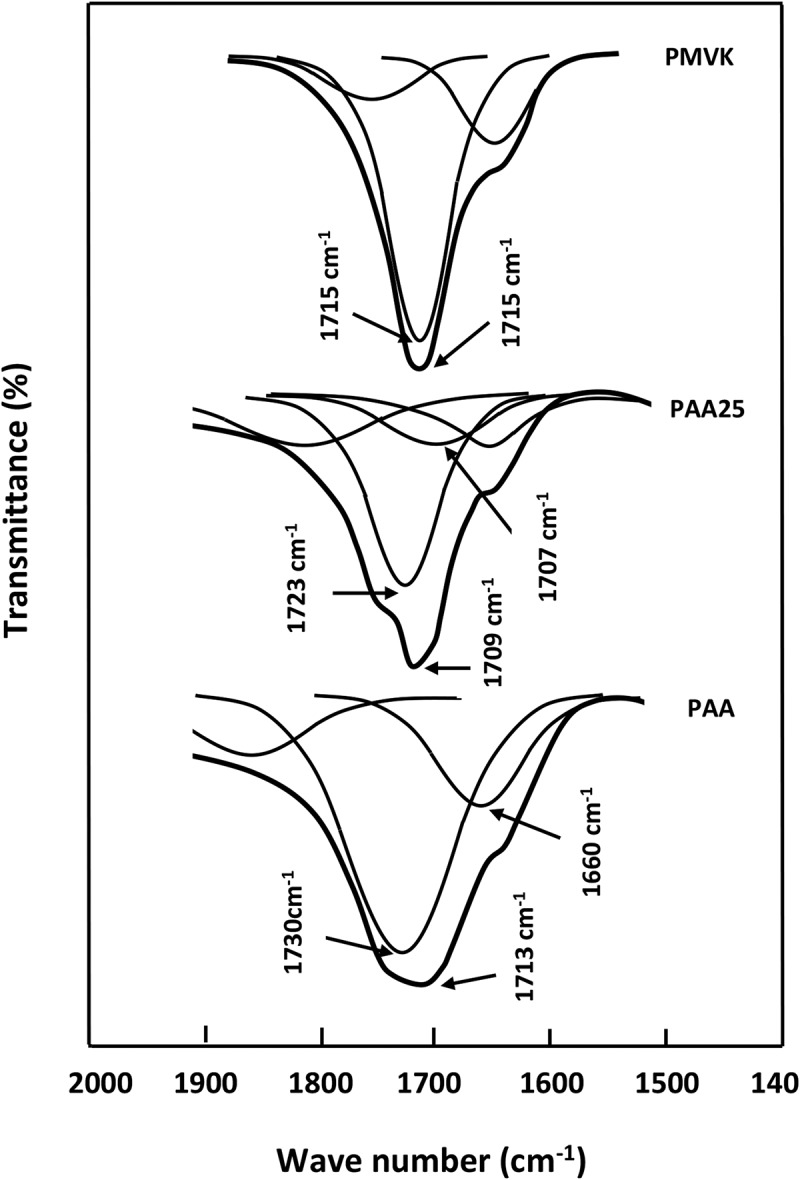
Deconvolution of the FTIR spectra of pure PAA, pure PMVK and PAA-25 blend in the carbonyl region.

**Figure 6.**
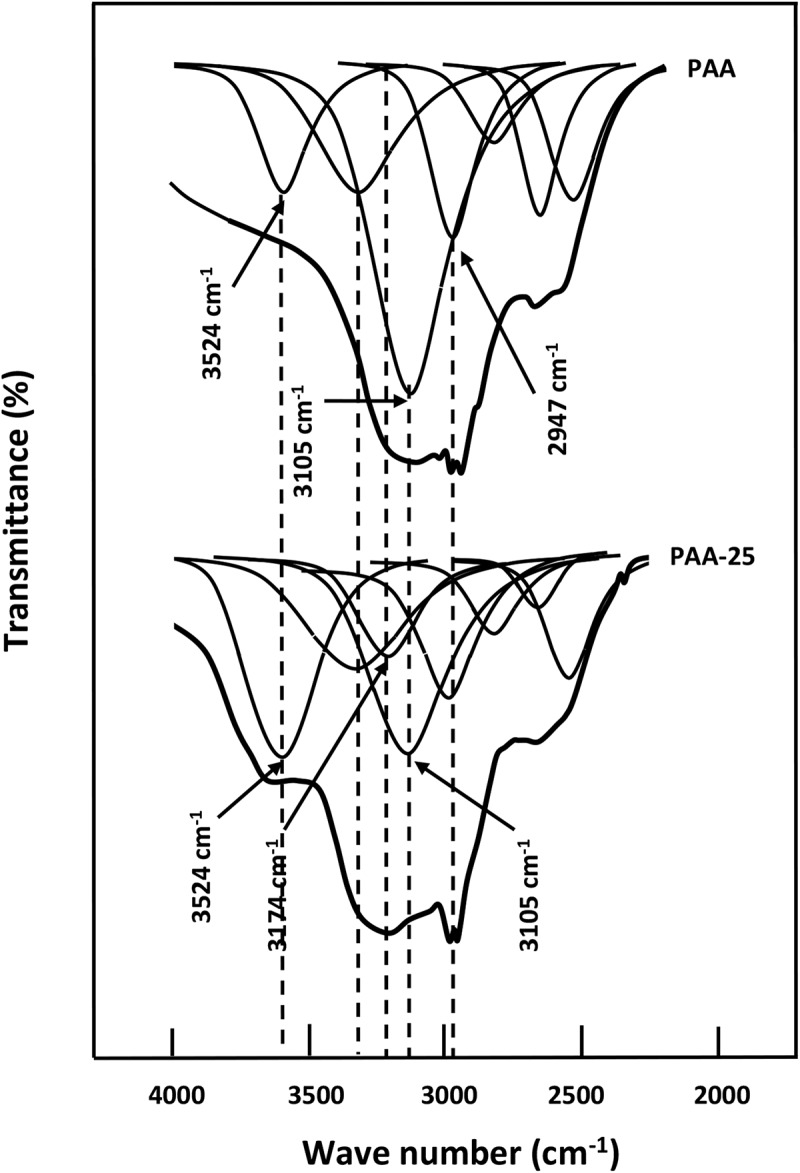
Deconvolution of the FTIR spectra of pure PAA, pure PMVK and PAA-25 blend in the hydroxyl region.

**Scheme 2.**
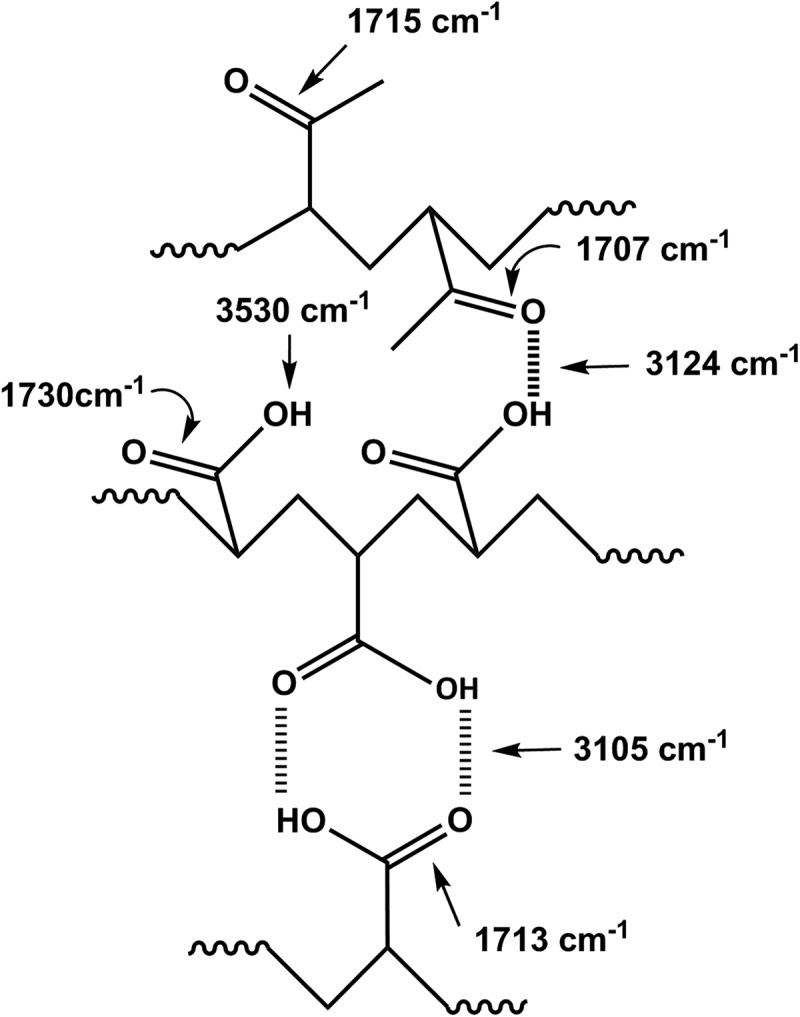
Hydrogen bonding between hydroxyl group of PAA and carbonyl group of PMVK in the PAA/PMVK blend.

The deconvolution of the PAA spectrum in lorentzian curves in this region, showed in Figure 5, unearths two principal peaks at 1730 and 1660 cm^−1^. These signals are assigned to C = O stretching for the AA units where the carbonyl groups are not hydrogen bonded and the conjugation with a C = C bond results in the delocalization of the π electrons of both unsaturated groups, respectively. On the other hand, the PMVK spectrum shows an absorption band at 1715 cm^−1^ attributed to the free (non-hydrogen-bonded) carbonyl stretching of the monomeric units. This corresponding peak shifts slightly toward the lower wave numbers when the PAA is incorporated in the PMVK matrix. This shift is probably caused by the apparition of a new absorption peak at 1707 cm^−1^ assigned to the associated carbonyls as shown for the PAA-25 in this same figure. This finding confirms the presence of hydrogen bonding between the carboxylic protons of PAA and the oxygen of the carbonyl of PMVK as shown in Scheme 2. The shift of the carbonyl band toward the lower wave numbers is attributed to the breaking of the moderate inter- and/or intra-molecular hydrogen bonds present in the pure PAA. In the hydroxyl region, Figure 4 shows the absorption broad band of pure PAA centered at 3215 cm^−1^. This band shifts toward the lower wave numbers (from 3215 to 3200 cm^−1^) when the concentration of PMVK in the blend is ranged between 0 and 25 wt%, then toward the higher wave numbers (from 3200 to 3253 cm^−1^) beyond. The deconvolution of the broad band in the hydroxyl region, showed in Figure 6, reveals for the pure PAA, three principal absorption bands at 3105, 3320 and 3524 cm^−1^ assigned to hydroxyl carboxylic dimers, water absorbed and free hydroxyl stretching vibration, respectively. The deconvoluted spectra of PAA/PMVK systems, in which the PAA-25 is taking as example, reveal a new absorption band at 3224 cm^−1^ attributed to the OH associated with the carbonyl group of PMVK.

The free and the associated carbonyl fractions in the blend are determined by Equation (3) [48–50] and the data obtained from the deconvolution spectra of the PAA/PMVK blends in the carbonyl region.

Here $A_{Asso}^{CO}$ and $A_{free}^{CO}$ are the band areas corresponding to the free and associated carbonyls, respectively. *a* is the absorptivity coefficient which is the specific absorption ratio of these bands (*a = a_ass_/a_free_*). In our case, we assume that *a_ass_* and *a_free_* have the same absorption coefficient, i.e., is equal to the unity. Table 3 summarizes the values of the free and associated carbonyl fractions for PAA/PMVK system with different compositions. The examination of these data reveals a slightly decrease of the associated carbonyl fraction as the PAA content in the blend increased.

**Table 3.** Curve fitting data from infrared spectra of PAA/PVMK blends in the 1650–1800 cm^−1^ region.

	Free C = O			Hydrogen bonded C = O		
Blend Composition PAA/PMVK	Frequency ν (cm^−1^)	Area$A_{free}^{CO}$	Fraction *f_free_*	Frequency ν (cm^−1^)	Area$A_{Asso}^{CO}$	Fraction *f_Asso_*
PAA-90	1715	5298	0.479	1711	5753	0.520
PAA-75	1718	5330	0.477	1705	5850	0.523
PAA-50	1720	5317	0.480	1705	5765	0.520
PAA-25	1723	5720	0.528	1707	5122	0.472
PAA-10	1727	6048	0.543	1712	5096	0.457

#### 3.1.4. XRD analysis

**Figure 7.**
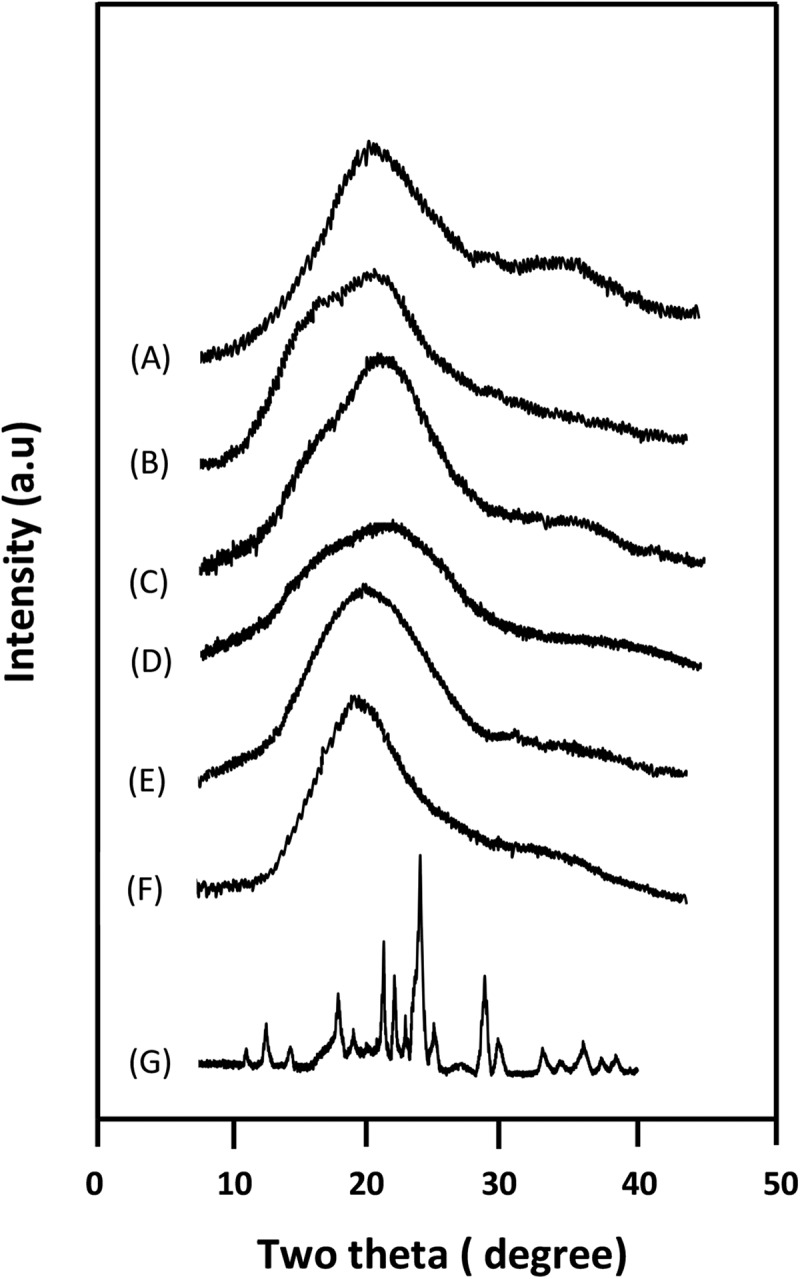
XRD patterns of pure STX, pure PAA, PAA50 blend and STX/PAA-50 system containing: A) 0 wt%; B) 3 wt%; C) 5 wt%; D) 7 wt% and E) 10 wt% of STX contents.

XRD analysis used in this work is to highlight the distribution mode of STX particles in the PAA/PMVK blend. The XRD patterns of pure components and STX/PAA5010 system containing 10 wt% of STX content, chosen as significant example, are featured in Figure 7. As can be seen from the STX spectrum, the crystalline structure of this medication is confirmed by the characteristic peaks centered at 13.3, 16.6, 21.0, 24.0, 28.2, 32.5 and 41.5 theta, which are fully agree with those of the literature [51,52]. While, the PAA and PMVK spectra as expected are devoid of any sharp signals that might indicates an eventual crystalline structure of these polymers. On the other hand, those of STX/PAA-50 system has practically the same patterns compared to that of PAA and PMVK revealing the complete disappearance of any peaks referred to the crystal structure of pure STX. This finding confirms the results obtained by the DSC analysis indicating that the STX incorporated in PAA-50 matrix is homogeneously distributed in its molecular state and not aggregated in its crystal forms.

### 3.2. Swelling behavior

**Figure 8.**
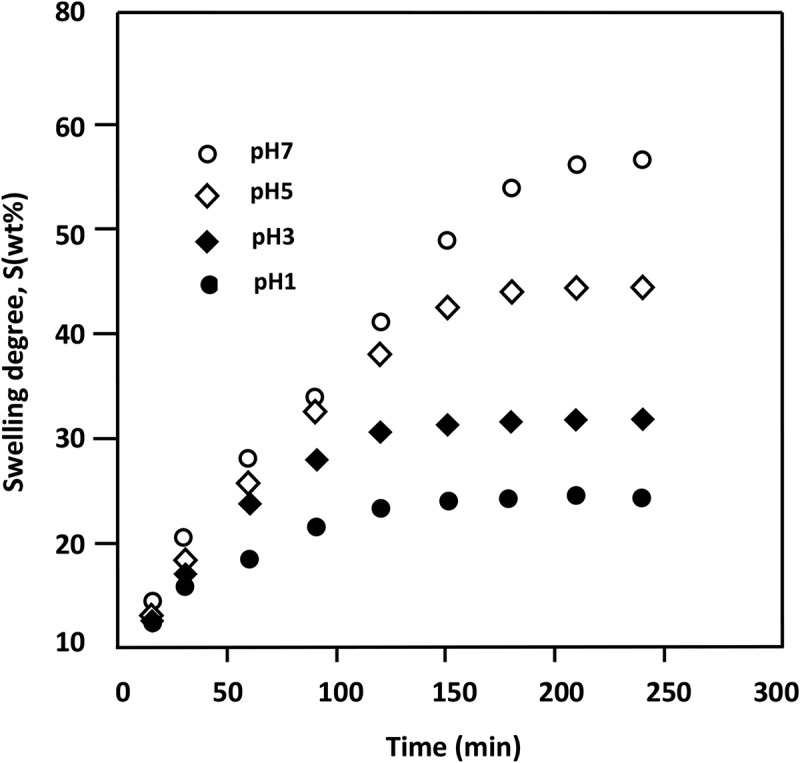
Variation of the swelling degree of PAA/PMVK hydrogels system in different media pHs.

Study the swelling behavior of a polymer hydrogel in water is indispensable to well understanding the diffusion of drug-water solution through a material used as carrier in the drug delivery domain. In this investigation, the capability of PMVK/PAA hydrogels to swell in water is performed at 37°C in different media pHs using Equation (3). The results of the swelling degree versus time for PAA5, taken as example, are plotted in Figure 8 and the values of the maximum swelling deducted are gathered in Table 4.

(3) $$S\left(\%\right)=\frac{w_{t}-w_{o}}{w_{o}}\times 100$$

**Table 4.** Swelling properties of water-PAA/PMVK hydrogels realized in different media pHs at 37°C. Noting that the *n* value was 0.5 for each composition and in any media pH.

PAA/PMVK Hydrogel Composition	*pH*	*S_∞_* *(wt%)*	*k*x10^2^ (min^−0.5^)	*D*x10^7^ (cm^2^.min^−1^)	PAA/PMVK hydrogel Composition	*pH*	*S_∞_* *(wt%)*	*k*x10^2^ (min^−0.5^)	*D*x10^7^ (cm^2^.min^−1^)
PAA-100	1	119.3	4.02	8.18	PAA-50	1	24.0	1.11	0.61
	3	134.7	4.56	10.53		3	31.3	1.35	0.91
	5	156.1	5.89	17.57		5	44.2	1.87	1.74
	7	178.6	6.18	19.34		7	56.8	2.52	3.17
PAA-90	1	68.3	2.18	2.20	PAA-25	1	20.1	0.83	0.27
	3	75.2	2.87	3.82		3	25.2	1.18	0.54
	5	87.2	3.56	5.89		5	29.6	1.29	0.65
	7	98.6	3.89	7.00		7	33.3	1.55	0.94
PAA-75	1	43.2	1.45	0.93	PAA-10	1	7.2	0.78	0.18
	3	56.4	1.78	1.41		3	9.4	0.97	0.28
	5	54.0	2.38	2.52		5	11.1	1.12	0.37
	7	72.5	2.89	3.71		7	14.7	1.27	0.48

where *w_o_* and *w_t_* are the film specimen weights before and at *t* time of the swelling process, respectively. These curve profiles reveal that the maximum swelling, S_∞_, corresponding to the swelling equilibrium is practically reached at about 3.5 h of the swelling process in media pH7 (56.8 wt%), 3.0 h in pH5(44.2 wt%), 2.15 h in pH3(31.3 wt%) and 2 h in pH1(24 wt%). The best performance for all PAA/PMVK hydrogels to swell in water at 37°C is observed in media pH7. Indeed, for example the water absorbed by these materials in neutral pH media varies from 1.44 to 2.37 times those in media pH1. It was also revealed from these data that the PAA-50 blend which contained equal polymer ratios has the best performance, because this system is capable to swell 2.37 times in neutral media pH that in pH1. This finding is desirable and constitutes as a key factor in the drug delivery domain since the majority of the drug incorporated in the polymer matrix must be released in intestines (pH7) and not in stomach (pH1).

To describe the dynamics of molecules through a polymer material during the swelling process, several mathematical models have been proposed by different authors based on the Fick’s law. These models are divided into three categories: Fickian diffusion, collective diffusion and non-Fickian diffusion models [53,54]. For many gels the mass transport of solvent or analyte molecules via convection mechanism should be also considered. The rate and extent of penetrant sorption into the polymer are determined by both concentration gradient-controlled and/or relaxation-controlled diffusion [55,56]. The diffusion categories of molecules into polymers when the maximum swelling degree is less or equal to 60 wt% are deducted from the exponent, *n*, of the following expression (Equation 4) [57–59]

(4) $$\frac{M_{t}}{M_{\infty }}=k\times t^{n}$$

where *M_t_* and *M_∞_* are the amount of molecules diffused into the polymer at time, *t*, and at equilibrium state (t = ∞), respectively. *n* is an exponent related to the type of diffusion mechanism. *k* is a constant related to diffusion coefficient, the structure and the film thickness of the polymer hydrogel. The exponent, *n*, and constant *k* can be deducted by plotting the data obtaining from the swelling process and Equation (4). According to Comyn [60], the kinetics that govern this dynamic, for the short times of the initial stage of diffusion and when the *M_t_/M_∞_* ratio is lower than 0.5 Equation (4), take the following equation:

(5) $$\frac{M_{t}}{M_{\infty }}=\frac{2}{l}\times {\left(\frac{D}{\pi }\right)}^{1/2}\times t^{1/2}$$

in which *D* can be deduced from the slope of the linear portion of the curve indicating the variation of *M_t_/M_∞_* versus square root of time. By analogy with Equation (4)*n = 0.5* and *k* takes the following expression:

(6) $$k=\frac{2}{l}{\left(\frac{D}{\pi }\right)}^{0.5}$$

**Figure 9.**
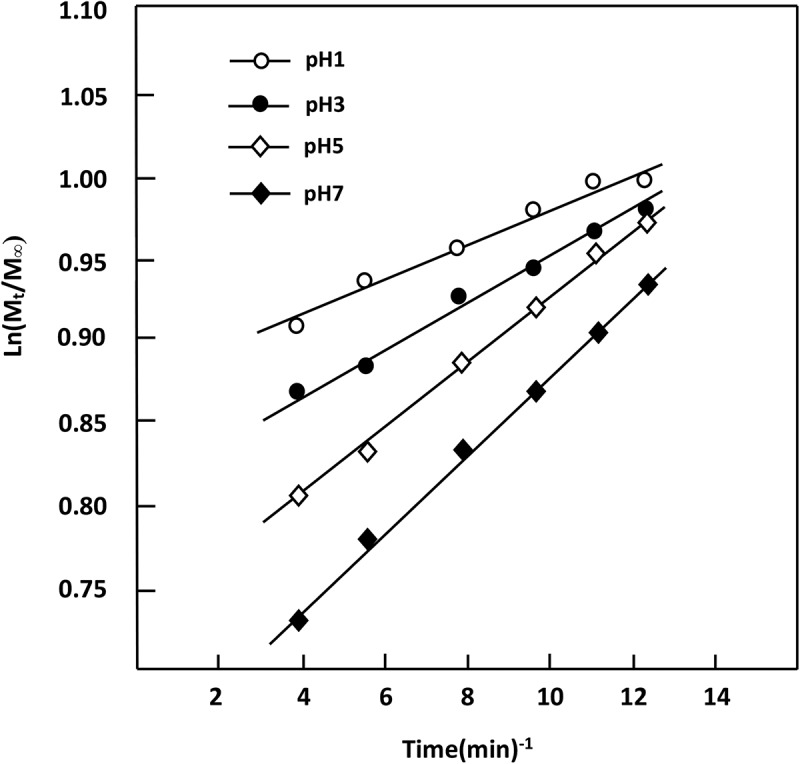
Variation of *Ln (M_t_/M_o_)* versus *Ln(t)* for the PAA-50 hydrogel.

As shown the example of the PAA-50 hydrogel in Figure 9, the variation of *M_t_/M_∞_* linearly varies with *t^1/2^* during the three first hours of the swelling process indicating that the diffusion of water molecules through the PAA/PMVK hydrogels obeys the Fick’s model as long as the temperature of this blend in media (37°C) is well above T_g_ (˞ 40°C).

This finding indicates that the diffusion of water through the polymer matrix is purely and simply governed by a mechanical process and non-disturbed by any other phenomena due to probable reaction between PAA and residual ethylene glycol used as crosslinking agent notably in acidic media. Noting that, the PAA in presence of a residual ethyleglycol in such media leads to increase the crosslinking degree of this polymer in the blend and consequently reduces the swelling degree. In general, for all PAA/PMVK compositions, the *D* and *k* values reported in Table 4 indicate that the diffusion rate slightly decreases as the pH media increased, thus indicating a low influence of the pH-media on the swelling process. In the drug delivery process the swelling degree of a material used as carrier must be moderated because higher swelling leads to a rapid release which can exceeded the maximum therapeutic level or sometimes leads to the destruction of the carrier. In opposite, a lower swelling leads to a slower release which cannot reach the therapeutic level.

### 3.3. Cytotoxicity test

**Figure 10.**
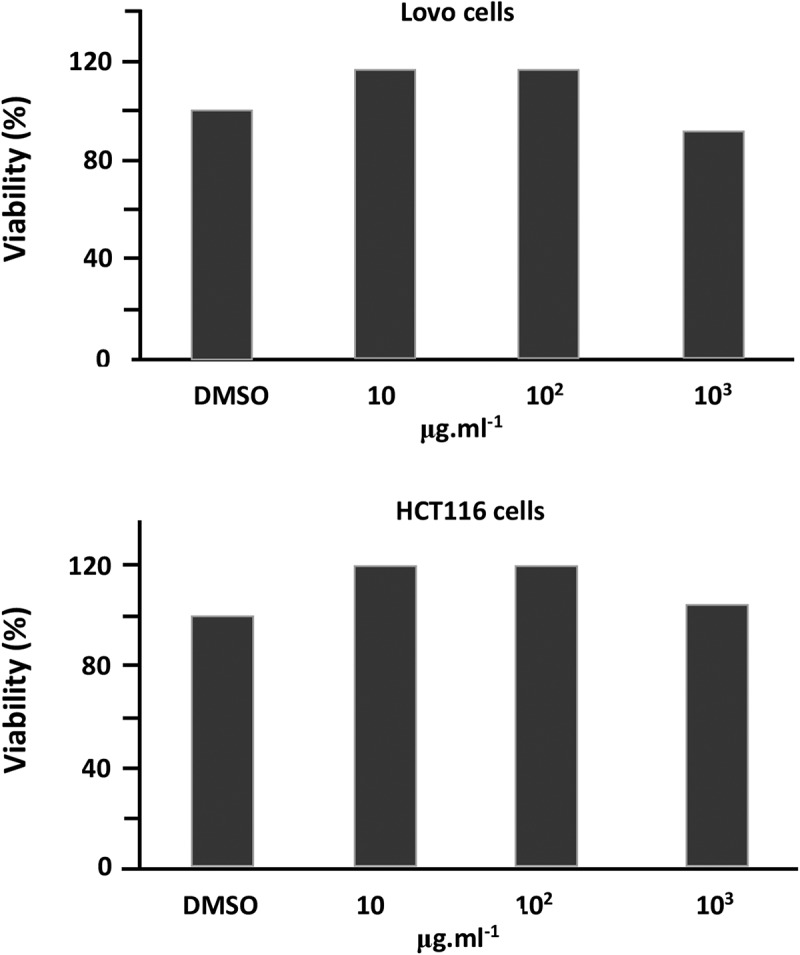
Cytotoxicity test of STX-10/PAA-50 hydrogel using the MTT assay at different concentrations.

The percentage viability of the cells is evaluated by applying Equation (7) [61] and the results obtained are grouped in Figure 10.

(7) $$Viability\left(\%\right)=\frac{D_{T}}{D_{C}}\times 100$$

According to the diagrams obtained, the cell viability is maximal using both methods, indicating that the PAA/PMVK hydrogels are out of the toxic level. These results also reveal that the percentage of viability slowly decrease when the concentration of polymer/KIT-5(150) increased and the best viability is observed at concentration ranged between 10 and 100 µg.ml^−1^.

From all previous studies, the results obtained converges toward the hydrogel containing equal amounts of polymers (PAA-50 hydrogel). Indeed, this system is proved as the adequate material for use as carrier in order to investigate the in vitro release dynamic of STX from the STX/PAA/PMVK hydrogel.

### 3.4. Drug solubility

The water-solubility of STX as any other therapeutically bioactive substance is also considered as a key factor in the drug release process. Indeed, this important parameter governs the dissolution of drug in the media then its absorption by the target organs. According to Petti et al. [62], the solubility of STX in water is estimated at 600 mg.L^−1^ and varied with pH media and temperature. Different researchers focused their investigations to enhance the solubility of this drug using different methods [63–65]. For example, the addition of β-cyclodextrin (β-CD) to the STX realized by Ozdemir et al. [64] using the phase solubility method permitted to enhance a maximum solubility of 188 mg.L^−1^ at pH7 and 175 mg.L^−1^ at media pH4.5. These same investigators by adding of EG to the previous mixture obtained 406 and 377 mg.L^−1^ in media pH7 and 4.5, respectively. Sekiguchi and Obi [65] demonstrated that the use of solid dispersion method by creating a eutectic mixture between the sulfonamide delivery and sulfathiazole aqueous solution permitted to obtain a solubility of 373 mg.L^−1^ at 25°C. In our investigation, the maximum solubility of STX reached from a cumulative release test obtained at 37°C during 73 h at different pH media is gathered in Table 5. As it can be seen that with STX-10/PAA-50 hydrogel, the maximum solubility of this drug is 371.36 ± 068 mg.L^−1^ at neutral media pH and 99.80 ± 08 mg.L^−1^at pH1.This finding confirms the results of the DSC and XRD analyses in which STX particles incorporated in the PMAA/PMVK matrix are found distributed in their molecules scale. Indeed, according to different investigations, the smaller the STX particle size, the higher is its solubility [66–68].

**Table 5.** Maximum STX amount dissolved in media at different pH during 73 h of the release process from PVADS materials at 37°C.

	STX amount dissolved (mg.L^−1^)			
pH media	**1**	**3**	**5**	**7**
System				
STX-3/PAA-50	24.56 ± 08	90.36 ± 09	114.48 ± 08	125.08 ± 06
STX-5/PAA-50	38.64 ± 09	173.40 ± 09	237.6 ± 08	207.24 ± 07
STX-7/PAA-50	44.40 ± 08	219.6 ± 07	285.28 ± 08	232.4 ± 08
STX-10/PAA-50	99.80 ± 08	301.04 ± 08	378.88 ± 08	371.36 ± 06

### 3.5. In vitro STX release

#### 3.5.1 Kinetics release of STX

**Figure 11.**
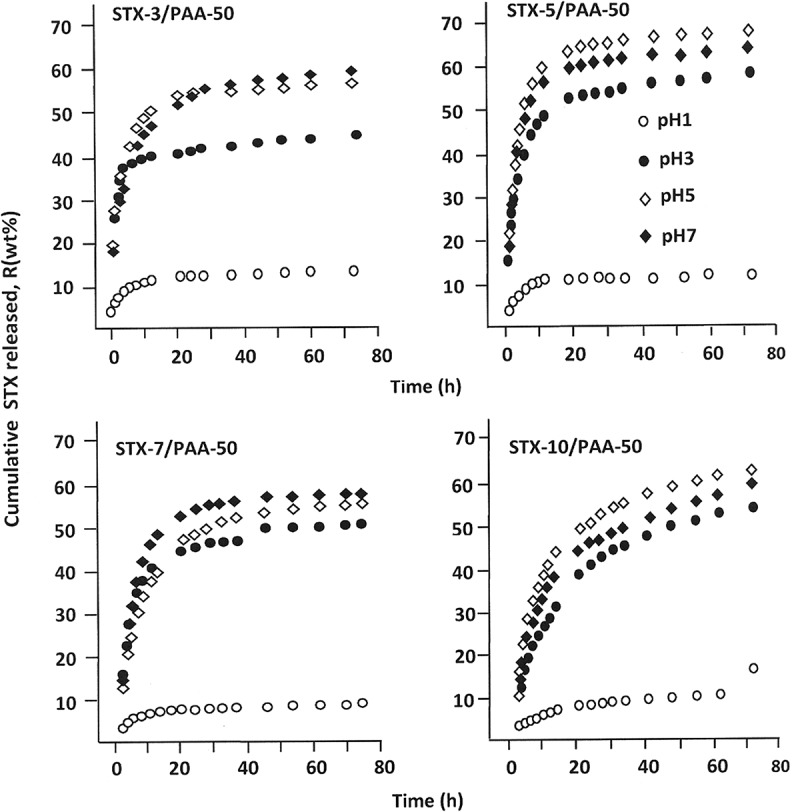
Cumulative STX released at 37°C from STX/PAA-50 hydrogel with different STX contents in different media pHs.

The cumulative release tests on STX/PAA-50 hydrogel containing 3, 5, 7 and 10 wt% of STX in different media pHs are performed at 37°C during 73 h and the results obtained using Equation (9) are plotted in Figure 11.

(9) $$R(wt\%)=\frac{m_{t}}{m_{o}}\times 100$$

where *m_o_* and *m_t_* are the initial mass of STX in the drug-carrier system and that at *t* time of the release process, respectively. As can be seen from these curves profiles that the percentage of STX released versus time followed a perfect logarithmic growth in which a considerable percentage of drug varied between 4 and 45 wt% of the total amount is released only during the 5 first hours of the release process depending on the initial STX amount incorporated in the drug-carrier system and the pH media. This phenomenon seems to be due to the high difference between the free enthalpy of dissolution (*ΔG_d_*) inside and outside the polymer matrix during this period until the equilibrium is reached. It is also observed that the best performance is reached with the system initially containing 5 wt% of STX (STX-5/PAA-50). Indeed, this system is capable to release more than 63 wt% of this medication throughout the process and during this same period, only 11.57 wt% has been released in media pH1. On the other hand, the lowest release dynamic of STX is obtained with the STX-10/PAA-50 hydrogel which is able to release a maximum of 49.91% by weight of STX in pH7 media and 13.49% by weight in pH1 media. This finding reflects the capacity of this system to dissolve this poorly soluble drug in different pH media.

#### 3.5.2. Diffusion behavior of STX

**Figure 12.**
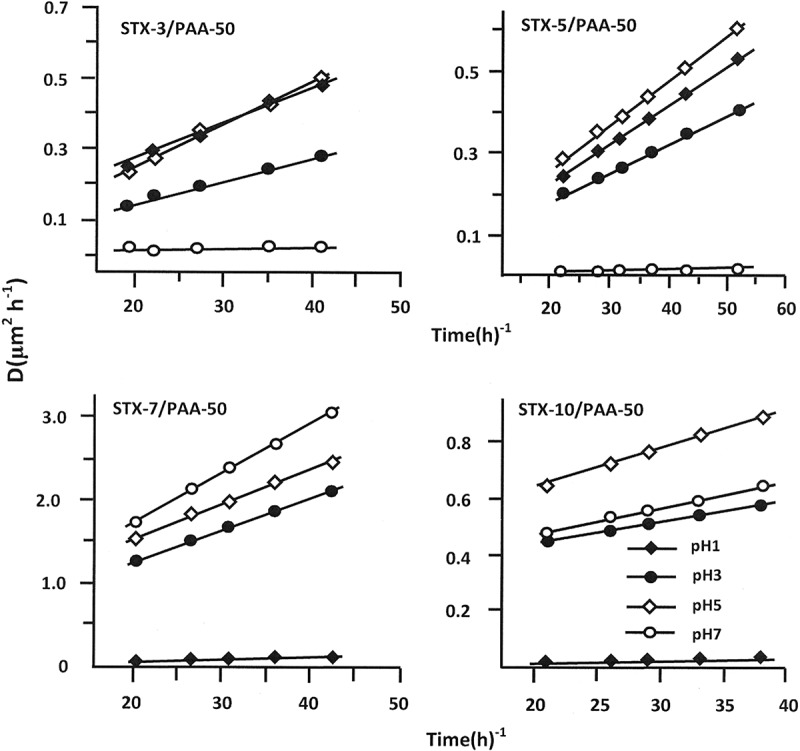
Diffusion coefficient of STX in different pH media from STX/PAA-50 hydrogel with different STX contents versus the inverse of time.

According to Lin et al. [69], the diffusion of a drug through a polymeric material obeys Fickian model if the cumulative drug released in a medium not exceed 60 wt% of the total drug amount incorporated in the polymer matrix. In this condition, the value of the diffusion coefficient, *D_STX_*, is calculated from Equation (10) [69–71].

(10) $$D_{STX}=\frac{0.196\times d^{2}}{t}{\left[\frac{m_{t}}{m_{o}}\right]}^{2}$$

where *d* is the thickness of the film specimen and *m_o_* and *m_t_* are defined as the mass of the STX before and at a certain time *t* of the release process, respectively. *D*_STX_ value is determined when the permanent regime is reached and therefore all STX particles deposed or pressed on the surface of the film surface are totally removed by washing in water. In these conditions, the curve profiles of *D_STZ_* versus time are meaningful and reflect exactly the dynamic of STX released in the media inside the polymer matrix. For the STX/PAA-50 system with different STX contents, the variation of *D_STX_* versus the inverse of time calculated from Equation (10) and the experimental data collected in different pH media are gathered in Figure 12. As can be observed from these curve profiles a straight line is obtained for each drug/carrier composition and in any pH medium. This finding indicates that the diffusion of STX through PAA/PMVK hydrogel obeys a fickian model for a release less than 60 wt% of the initial STX amount. This finding also indicates that the permanent regime of the dynamic release is reached. On the light of these results, it was possible to build our investigation on the second zone of the release process which is generally localized between 20 and 73 h.

#### 3.5.3 Effect of the initial STX amount

**Figure 13.**
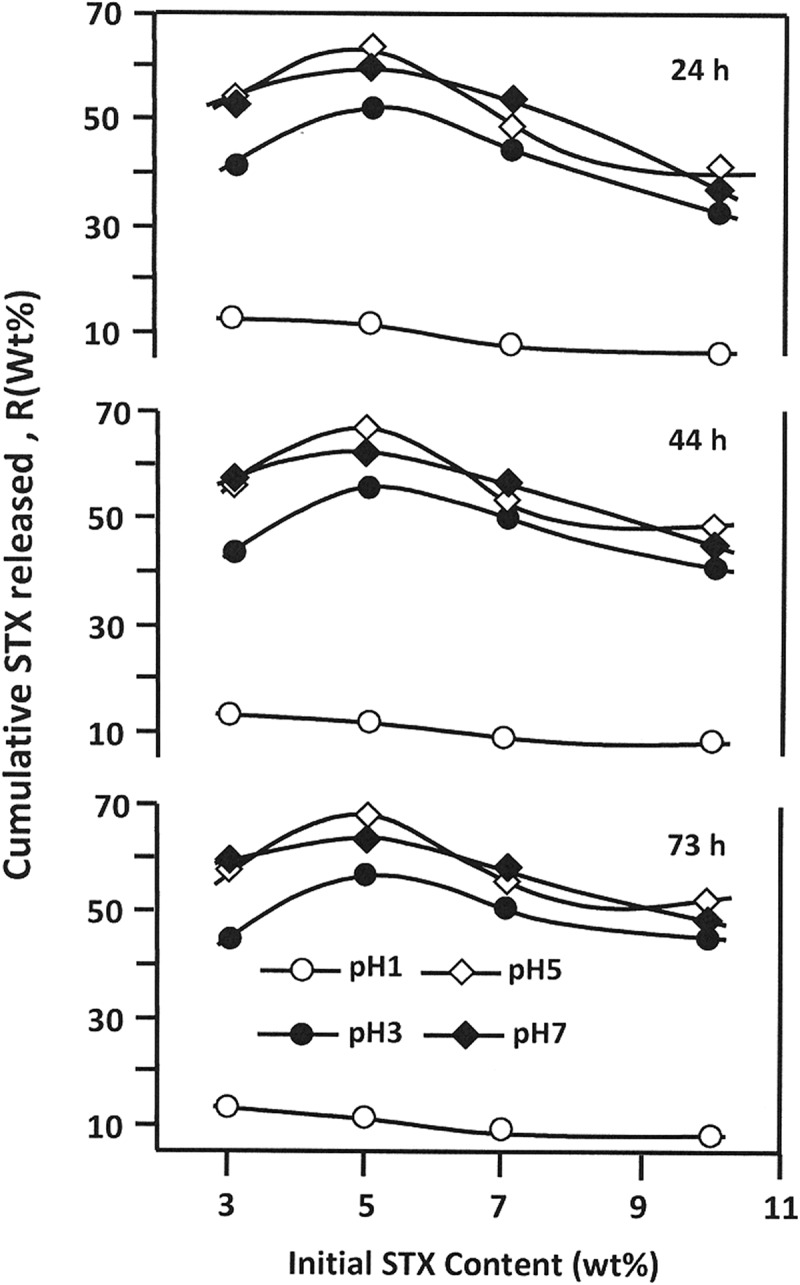
Cumulative STX amount released from STX/PAA-50 hydrogel with different STX contents during 23, 44 and 73 h versus the initial contents in different media pHs.

Figure 13 shows the influence of the initial STX amount incorporated in PAA/PMVK carrier on the dynamic release of STX at different media pHs at 23, 44 and 73h of release process, in which these periods correspond to the average intestinal transit times (AITT) [72,73]. From these data, it is revealed that the release dynamic of STX varies following a same trend for all durations and media pHs. For all specimens, the cumulative STX released reaches a maximum at 5 wt% of STX in the polymer matrix except that in media pH1 in which the release of this drug continues to decrease slowly in this composition range. This phenomenon can be principally attributed to the solubility of STX in the media, because it is obvious that 3 wt% of STX in the polymer matrix is much easier to be dissolved in water than 10 wt%. The low release dynamic observed in media pH1 is probably caused by an eventual reduction of the swelling degree of the PAA/PMVK hydrogel and the high solubility of STX observed in neutral media as previously revealed through the results plotted in Figure 13. The low release dynamic observed in media pH1 will be more explained in the effect of the swelling degree (Section 3.9.4).

#### 3.5.4 Effect of pH media

**Figure 14.**
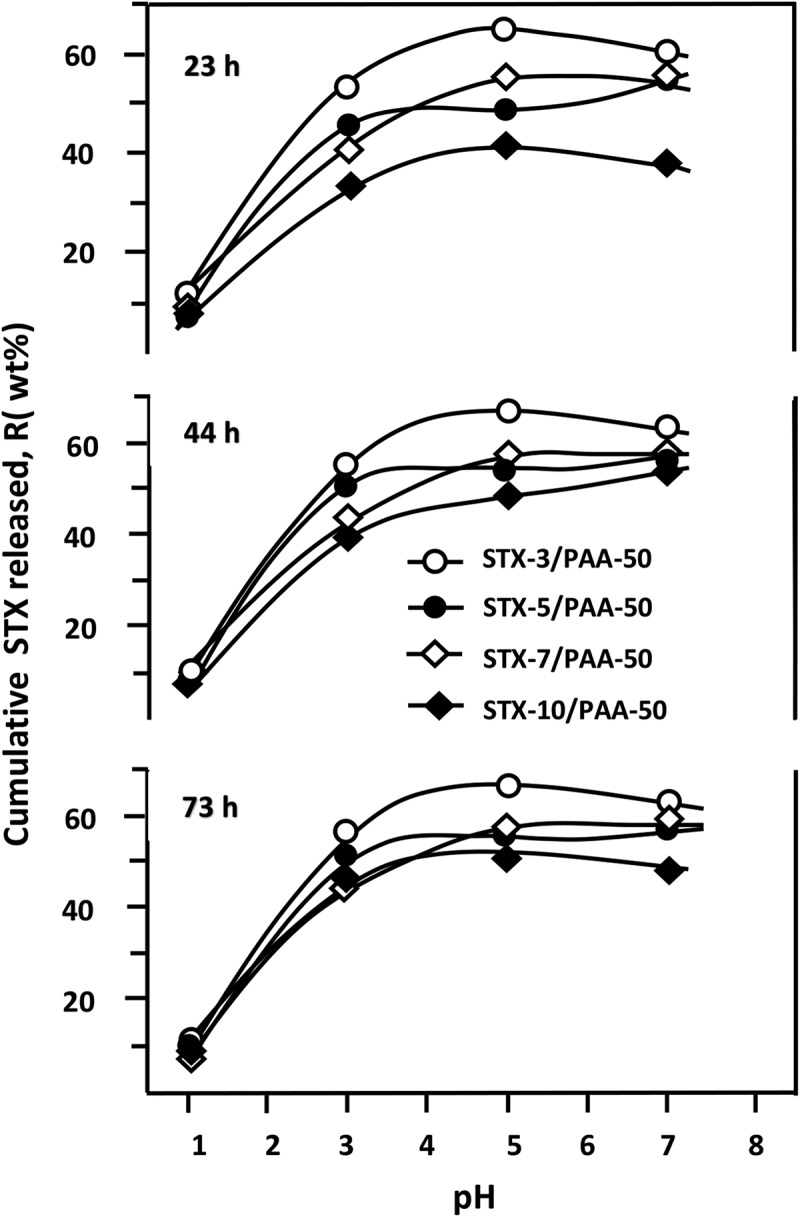
Influence of pH media on the release dynamic of STX from STX/PAA-50 hydrogel with different STX contents during 23, 44 and 73 h of the release process.

The variations of the cumulative STX released from STX/PAA-50 systems versus pH media during 23, 44 and 73 h of the release process are gathered in Figure 14. From these curve profiles it is revealed that the release dynamics of STX from the STX/PAA-50 specimens have practically the same trends at any duration and drug/carrier composition in which the cumulative drug released dramatically increases to reach a maximum in media pH5 and slowly decreases beyond, except that of STX-5/PAA-50 which reaches its maximum in pH4 then a slight minimum in media pH5. The release dynamic of STX from the STX −10/PAA-50 specimen, during the period of 44 h, continues to increase slowly in media pH superior to 5. As explained in the previous paragraph, this finding seems to be directly linked to the solubility of STX in the media, in which the STX released dramatically increases as the media pH increased in the investigated range.

#### 3.5.5 Influence of the swelling degree

**Figure 15.**
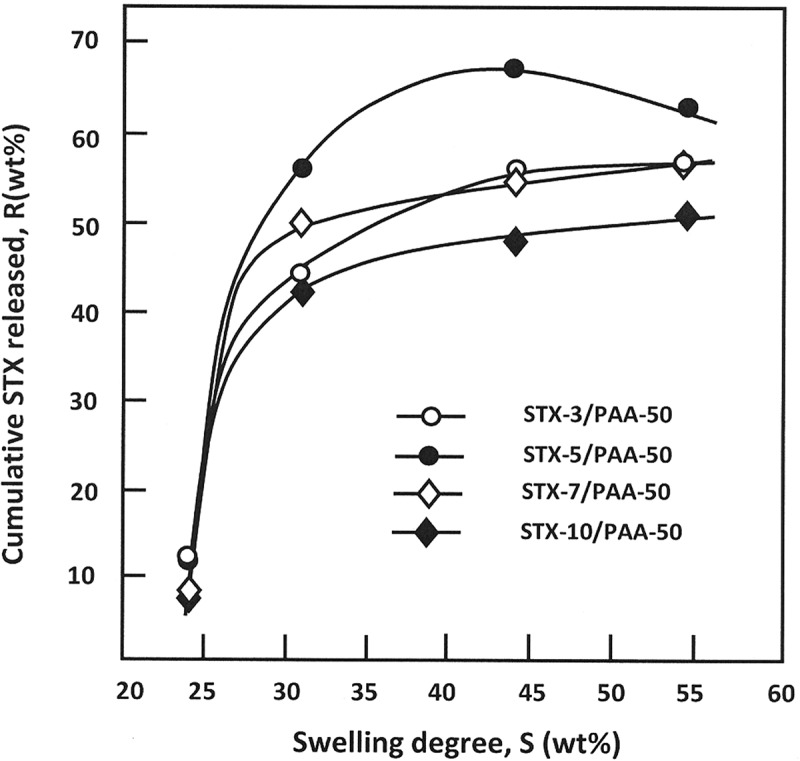
Influence of the swelling degree of the PAA/PMVK hydrogel on the release dynamic of STX from STX/PAA-50 hydrogel with different STX contents.

The variation of the total STX released from the STX/PAA-50 hydrogels taken at 44 h of the release process versus the swelling degree of PAA50 hydrogel is shown in Figure 15. It is revealed from these curve profiles the release dynamic of STX versus the swelling degree have practically the same trends. The release dynamic of STX dramatically increases when the swelling degree is inferior to 30 wt%, then slowly increases beyond, except those for the specimen containing 5 wt% of STX, in which the dynamic release reaches a maximum at a swelling degree of 44.2 wt% and that containing 3 wt% stabilizes before this value. The increase of the cumulative STX with the swelling degree seems to be evident, because increasing water amount in the material leads to increase the diffusion factor inside the polymer matrix as showed previously in Table 3 and consequently favorites the dissolution of STX. The slowdown of the release dynamic observed when the swelling degree is superior to 30 wt% is probably governed by the equilibrium of the STX-water solubility inside and outside the polymer matrix. However, the decrease of the cumulative drug released beyond 44.2 wt%, observed in case of STX-5/PAA-50, is probably due to the depletion of the STX stored inside the drug carrier system. In addition to the water-polymer interactions, other important parameters such as drug-polymer and drug-media interactions can also be involved to orient the dynamic release of a drug from a drug-carrier system.

#### 3.5.6 SEM analysis

**Figure 16.**
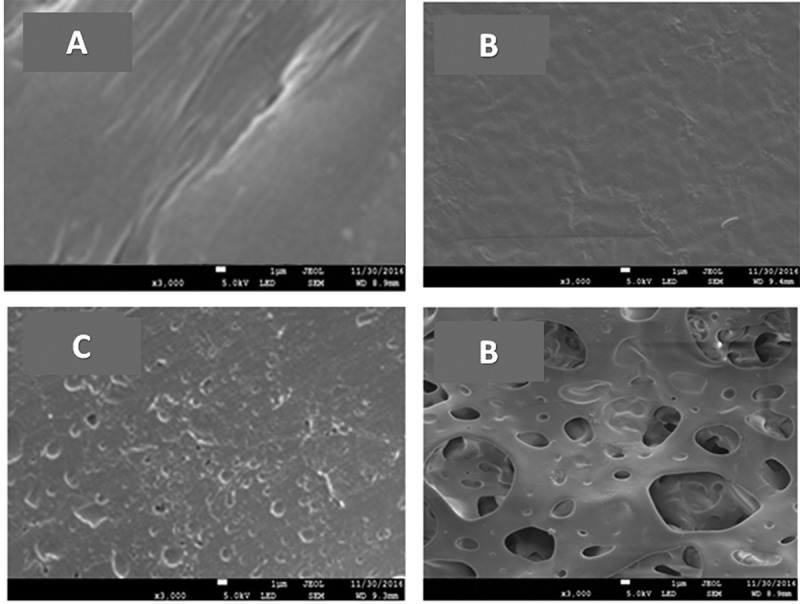
SEM micrographs of PAA/PMVK hydrogel (A), STX/PAA-50 hydrogel before (B) and after the release process in media pH1 (C) and pH7 (D).

The SEM micrographs of PAA50 and STX-10/PAA-50 specimens before and after the release process in media pH1 and 7 taken as significant examples are presented in Figure 16. As can be seen from the PAA-50 (image-A) a smooth and uniform surface morphology free from any particles aggregated or heterogenic zones that may indicate the miscibility of this pair of polymers. The image of STX-10/PAA-50 before the release process (image-B) shows homogeny surface morphology, less clear compared to that of the polymer blend looking like the sea-water waves in bad weather. This situation seems to be due to the different constraints caused by the combination of different interactions between the components of the drug-carrier systems such as those between PAA and STX (acid-base) and PMVK and STX (Shiff base). This observation confirms the perfect homogeneity of these systems in their molecular state which was previously proved by DSC and XRD analysis. The STX/PAA-50 micrographs taken after the release process in pH 1 (image-C) and 7 (image-D) media show erosion characterized by typical cavities, grooves and pores occupied initially by the STX molecules. As illustrated in these images, these cavities are more marked when the release is carried in neutral media pH indicating that a large amount of STX is released in this media compared to that released in pH1 media, thus confirming the higher release performance obtained in neutral pH media.

#### 3.5.7 Performance of *STX/PAA50* drug-carrier system

The kinetic of STX released undertaken in this work is based on the data of the instantaneous release rates of this drug from STX/PAA-50 drug-carrier system. The slopes of the linear portions of the kinetic curves showed in Figure 11 reveal the presence of two principal stable zones for each drug- carrier composition as shown in Table 6. For example, the STX-3/PAA-50 system presents in media pH7 its first zone which is short (4 h) and characterized by a cumulative STX released of 7.0 ± 0.5 wt% with a release rate of 1.75 ± 0.04 wt.h^−1^. This step is followed by a larger zone localized between 30 and 73 h in which 4.0 ± 0.8 wt% of the total STX amount is released with a release rate of 0.09 ± 0.03 wt%.h^−1^. According to the criteria based on the maximum drug amount uniformly released in intestinal media (PH≈ 7) with an adequate release rate during a longest period, it is revealed that the best performance is attributed to the STX-10/PAA-50 system. Indeed, this system is capable to release uniformly the maximum percentage of STX in neutral pH media which is 18.0 wt% during the longest period (50 h) with a moderated release rate of 0.34 wt%.h^−1^ and only 8.0 wt% in media pH1 (stomach pH) during a period of 15 h with a release rate of 0.53 wt%.h^−1^. According to the data of the average stomach transit [74], only from 0.53 to 2.13 wt% of STX will be released in the stomach, in which the transit is averaged between 1 and 4 h. However, in the intestines, in which the average transit is ranged between 4 and 72 h, more than 1.44 and 18.0 wt% of this drug will be absorbed, respectively.

**Table 6.** Stable zones and instantaneous release rate of STX from STX/PAA-50 drug carrier systems at 37°C and in different media pHs.

System	pH	Stable zone (h)	STX released (wt %)	Release rate (wt %/h)	System	Stable zone (h)	STX released (wt %)	Release rate (wt %/h)
**STX-3/PAA-50**	1	0–2 10–73	8.0 ± 0.2 2.5 ± 0.5	4.00 ± 0.04 0.04 ± 0.03	**STX-5/PAA-50**	0–9 10–73	10.0 ± 0.8 2.0 ± 0.3	1.11 ± 0.02 0.03 ± 0.02
	3	0–3 8–73	38.0 ± 0.2 6.0 ± 0.5	12.66 ± 0.04 0.09 ± 0.01		0–7 20–73	48.0 ± 0.6 7.0 ± 0.6	6.86 ± 0.04 0.14 ± 0.02
	5	0–6 20–73	42.0 ± 0.6 2.0 ± 0.7	7.00 ± 0.08 0.04 ± 0.04		0–7 20–73	47.0 ± 0.6 6.0 ± 0.5	6.71 ± 0.05 0.12 ± 0.03
	7	0–4 30–73	7.0 ± 0.5 4.0 ± 0.8	1.75 ± 0.04 0.09 ± 0.03		0–4 20–73	40.0 ± 0.4 4.5 ± 0.5	10.00 ± 0.10 0.09 ± 0.03
**STX-7/PAA-50**	1	0–10 10–73	6.0 ± 0.3 3.0 ± 0.4	0.60 ± 0.02 0.06 ± 0.02	**STX-10/PAA-50**	0–15 20–73	8.0 ± 0.3 2.5 ± 0.3	0.53 ± 0.03 0.05 ± 0.04
	3	0–10 20–73	35.0 ± 0.3 6.0 ± 0.5	3.40 ± 0.02 0.11 ± 0.02		0–16 24–73	35.0 ± 0.4 13.0 ± 0.4	2.19 ± 0.04 0.02 ± 0.01
	5	0–10 28–73	40.0 ± 0.4 8.0 ± 0.8	4.00 ± 0.03 0.18 ± 0.03		0–10 23–73	37.0 ± 0.5 13.0 ± 0.5	3.70 ± 0.05 0.26 ± 0.03
	7	0–10 27–73	47.0 ± 0.4 5.0 ± 0.5	4.70 ± 0.02 0.11 ± 0.02		0–13 20–73	38.0 ± 0.5 18.0 ± 0.5	2.92 ± 0.03 0.34 ± 0.02

According to Belzer et al. [75], the average of the total gastrointestinal transit time (GITT) is ranged between 53 and 88 h and distributed on three principal steps, which are the transit time in stomach (1–4 h at pH ≈ 1.5 – 3.5), the small intestinal transit (4 – 12 h at pH ≈ 7 – 9) and the transit time in the colon (48 – 72 h at pH ≈ 5 – 7). Taking into account the pH media and the transit times in different digestive organs the distribution of the in vitro cumulative STX released, regardless of any enzymes and microorganism actions, is investigated and the estimated results obtained are gathered in Table 6. Based upon the criteria that stipule that a best performance of a drug-carrier system is obtained when the highest amount of drug is uniformly released in the intestines (neutral media pH) during the longest time and the lower amount of the drug released in stomach (in acidic media) during the shortest time. By simple calculation using the release curves and the data of the average total gastrointestinal transit time (GITT) estimated by Belzer et al, it was possible to estimate the distribution of STX released in these different organs and the R(wt%) intestines/R(wt%) stomach ratio and the results obtained are grouped in Table 7. From these data, it is revealed that the STX-10/PAA-50 drug-carrier system is considered as the best performing compared to the other systems, because it is capable to reach the higher R(wt%) intestines/R(wt%) stomach ratio with 32.3 for the lowest transit and 11.5 for the highest transit. This finding indicates that this drug-carrier system which initially contained 10 wt% of drug is capable to deliver in the intestines an amount of STX equal between 11.5 and 32.5 folds that delivered in the stomach.

**Table 7.** Estimated distribution of the cumulative STX released from STX/PAA-50 drug-carrier systems on the principal digestive organs.

STX/PAA-50 Composition	Stomach transit (wt%)		Small intestine transit (wt%)		Colon transit (wt%)		Intest/Stomach ratio (wt/wt)	
Transit time	Min (1h)	Max (4h)	Min (4h)	Max (12h)	Min (48h)	Max (72 h)	Min	Max
STX-3/PAA-50	19.0	23.5	40. 0	49.0	41.0	27.5	4.26	3.26
STX-5/PAA-50	16.5	23.0	45.0	58.0	38.5	19.0	5.06	3.35
STX-7/PAA-50	4.8	16.5	28.0	51.0	67.3	33.0	19.9	5.09
STX-10/PAA-50	3.0	8.0	24.0	41	73.0	51.0	32.3	11.5

## 4. Conclusion

PAA is a weak polyelectrolyte and has been used in several biomedical domains such as immunology, drug delivery and enzyme immobilization. Carboxylic acid substituent groups of this polymer enable further modifications, and for drug/biomolecule binding in mild conditions without any structural deterioration. However, the high swelling in water of this polymer limits its utilization in a wide range of the biomedical applications. In this work, mixing PAA with PMVK in a miscible blend can be an alternative to resolve this inconvenient. Indeed, the miscibility of PAA/PMVK is effectively proved by DSC and FTIR methods The DSC, XRD and SEM analyses revealed that the STX particles incorporated in the blend by solvent casting are uniformly distributed in the polymer matrix in their molecules state. The cytotoxicity, and the cell adhesion and cell growth tests indicated that the PAA/PMVK hydrogels are out of the toxic level. The swelling study of the prepared materials revealed that the swelling degree of this hydrogel can be easily controlled by addition of an adequate PMVK amount to the PAA matrix, in which the specimen with equal PAA/PMVK ratio was the more performing and desirable in the drug delivery field. The water solubility test of STX by its simple incorporation in the PAA-50 using the solvent casting method is considered as an adequate technique which can resolve many problems related to its absorption and bioavailability, thus facilitating the increase of the therapeutic plasma concentrations after oral administration. Indeed, this technique reveals the dissolution of more than 371 mg.L^−1^ of STX at neutral media. The mass transfer study revealed that the diffusion phenomenon of STX through PAA50 hydrogel obeys a fickian model. The in vitro release dynamic of this medication from the STX/PAA-50 hydrogels revealed that the best performance is reached with that initially contained 10 wt% of STX content. Indeed, according to According to the total gastrointestinal transit time estimated by Belzer et al, the estimate distribution of STX released in these different organs indicated that the best R(wt%) intestines/R(wt%) stomach ratio is obtained with STX-10/PAA-50 drug-carrier system indicating that this drug-carrier system is capable to deliver in the intestines an amount of STX equal between 11.5 and 32.5 folds that delivered in the stomach.

## References

[1] Nair LS, Laurencin CT. Biodegradable polymers as biomaterials. Prog Polym Sci. 2007;32(8–9):762–798.

[2] Hofmann G. Biodegradable implants in orthopaedic surgery—a review on the state-of-the-art. Clinic Mater. 1992;10(1–2):75–80. 10.1016/0267-6605(92)90088-b 10171206

[3] Mackenzie PJ, Schertzer RM, Isbister CM Comparison of silicone and polypropylene Ahmed glaucoma valves: two-year follow-up. Canad J Ophthalmol. 2007;42(2):227–232. 17392844

[4] Bellucci R An introduction to intraocular lenses: material, optics, haptics, design and aberration. In: Güell JL, editor. Cataract. Vol. 3 Basel: Karger Publishers; 2013 p. 38–55.

[5] Leggat PA, Smith DR, Kedjarune U Surgical applications of methyl methacrylate: a review of toxicity. Arch Envir Occup Health. 2009;64(3):207–212. 10.1080/19338240903241291 19864224

[6] Tanaka M, Mochizuki A Clarification of the blood compatibility mechanism by controlling the water structure at the blood–poly (meth) acrylate interface. J Biomater Sci Polym Ed. 2010;21(14):1849–1863. 20699056 10.1163/092050610X517220

[7] Tanaka M, Motomura T, Kawada M, et al Blood compatible aspects of poly (2-methoxyethylacrylate)(PMEA)—relationship between protein adsorption and platelet adhesion on PMEA surface. Biomaterials. 2000;21(14):1471–1481. 10872776 10.1016/s0142-9612(00)00031-4

[8] Li S Hydrolytic degradation characteristics of aliphatic polyesters derived from lactic and glycolic acids. J Biomed Mater Res. 1999;48(3):342–353. 10398040 10.1002/(sici)1097-4636(1999)48:3<342::aid-jbm20>3.0.co;2-7

[9] Olabisi O, Robeson L, Shaw M Polymer-polymer miscibility. New York: Academic press; 1979.

[10] Albertsson A-C, Eklund M Synthesis of copolymers of 1, 3‐dioxan‐2‐one and oxepan‐2‐one using coordination catalysts. J Polym Sci Part A Polym Chem. 1994;32(2):265–279.

[11] Parameswaranpillai J, Thomas S, Grohens Y. Polymer blends: state of the art, new challenges, and opportunities. In: Thomas S, Grohens Y, Jyotishkumar P, editors. Characterization of polymer blends: miscibility, morphology and interfaces. Germany: Wiley-VCH Verlag GmbH & Co. KGaA- Weinheim; 2015. p. 1–6.

[12] Ding J, Zhuang X, Xiao C, et al Preparation of photo-cross-linked pH-responsive polypeptide nanogels as potential carriers for controlled drug delivery. J Mater Chem. 2011;21(30):11383–11391.

[13] Tang H, Tsarevsky-Nicolay V Preparation and functionalization of linear and reductively degradable highly branched cyanoacrylate-based polymers. J Polym Sci Part A Polym Chem. 2016;54(23):3683–3693.

[14] Alotaibi NM, Aouak T Preparation and non isothermal crystallization kinetic of acetylsalicylic acid-poly(vinylalcohol-co-ethylene) blend. Application in drug delivery domain. Macromol Res. 2013;21(7):747–756.

[15] Chernga JY, Houa TY, Shihb MF, et al Polyurethane-based drug delivery systems. Int J Pharmaceut. 2013;450(1–2):145–162. 10.1016/j.ijpharm.2013.04.063 23632262

[16] Ahuja G, Pathak K Porous carriers for controlled/modulated drug delivery. Indian J Pharm Sci. 2009;71(6):599–607. 20376211 10.4103/0250-474X.59540 PMC2846463

[17] Kadajji VG, Betageri GV Water soluble polymers for pharmaceutical applications. Polymers (Basel). 2011;3(4):1972–2009.

[18] Bromberg L Polyether-modified poly(acrylic acid): synthesis and applications. Ind Eng Chem Res. 1998;37(11):4267–4274.

[19] de Gigglio E, Cafaga D, Ricci MA, et al Biocompatibility of poly(acrylic acid) thin coatings electro-synthesized onto TiAlV-based implants, J. Bioact Compat Polym. 2010;25(4):374–391.

[20] Dehbari N, Tavakoli J, Khatrao SS, et al In situ polymerized hyperbranched polymer reinforced poly(acrylic acid) hydrogels. Mater Chem Front. 2017;1(10):1995–2004.

[21] Morton SW, Shah NJ, Quadir MA, et al Osteotropic therapy via targeted layer-by-layer nanoparticles. Adv Healthc Mater. 2014;3(6):867–875. 24124132 10.1002/adhm.201300465 PMC4041853

[22] Ürkmez AŞ, Bayir E, Bilgi E, et al Biocompatible polymeric coatings do not inherently reduce the cytotoxicity of iron oxide nanoparticles. Turk J Biol. 2017;41:322–332.

[23] Madsen F, Peppas NA Complexation graft copolymer networks: swelling properties, calcium binding and proteolytic enzyme inhibition. Biomaterials. 1999;20(18):1701–1708. 10503971 10.1016/s0142-9612(99)00071-x

[24] Gao X, He C, Xiao C, et al Biodegradable pH-responsive polyacrylic acid derivative hydrogels with tunable swelling behavior for oral delivery of insulin. Polymer (Guildf). 2013;54(7):1786–1793.

[25] Calixto G, Yoshii AC, Rocha E Silva H, et al Polyacrylic acid polymers hydrogels intended to topical drug delivery: preparation and characterization. Pharm Dev Technol. 2015;20(4):490–496. 25975700 10.3109/10837450.2014.882941

[26] Wang H, Dai T, Zhou S, et al Self-assembly assisted fabrication of dextran-based nanohydrogels with reduction-cleavable junctions for applications as efficient drug delivery systems. Sci Rep. 2017;7:40011. 28071743 10.1038/srep40011 PMC5223173

[27] Carvalho FC, Calixto G, Hatakeyama IN, et al Rheological, mechanical, and bioadhesive behavior of hydrogels to optimize skin delivery systems. Drug Devel Ind Pharm. 2013;39(11):1750–1757. 23216218 10.3109/03639045.2012.734510

[28] Larsson M, Gustafsson S, Olsson E, et al Effect of calcium neutralization on elastic and swelling properties of crosslinked poly (acrylic acid)-correlation to inhomogeneities and phase behaviour. e-Polymers. 2009;9(1):1618–7229.

[29] Müller C, Leithner K, Hauptstein S, et al Preparation and characterization of mucus-penetrating papain/poly (acrylic acid) nanoparticles for oral drug delivery applications. J Nanopart Res. 2013;15(1):1353.

[30] Larsson M, Bergstrand A, Mesiah L, et al Nanocomposites of polyacrylic acid nanogels and biodegradable polyhydroxybutyrate for bone regeneration and drug delivery. J Nanomater. 2014;2014:1–9.

[31] Guo Y, Sun J, Bai S, et al pH-Sensitive performance of dextran–poly (acrylic acid) copolymer and its application in controlled in vitro release of ibuprofen. Int J Polym Mater Polym Biomater. 2017;66(17):900–906.

[32] Shabir F, Erum A, Tulain UR, et al Preparation and characterization of pH sensitive crosslinked Linseed polysaccharides-co-acrylic acid/methacrylic acid hydrogels for controlled delivery of ketoprofen. Des Monom Polym. 2017;20(1):485–495. 10.1080/15685551.2017.1368116 PMC5784885 29491820

[33] Foltzer MA, Reese RE Trimethoprim-sulfamethoxazole and other sulfonamides. Med Clin North Am. 1987;71(6):1177–1194. 3320619 10.1016/s0025-7125(16)30805-7

[34] Gutman LT The use of trimethoprim-sulfamethoxazole in children: a review of adverse reactions and indications. Pediatr Infect Dis J. 1984;3(4):349–357. 10.1097/00006454-198407000-00018 6473140

[35] Rieder MJ, King SM, Read S Adverse reactions to trimethoprim-sulfamethoxazole among children with human immunodeficiency virus infection. Pediatr Infect Dis J. 1997;16(11):1028–1031. 9384334 10.1097/00006454-199711000-00005

[36] Hussain Z, Yousif E, Ahmed A, et al Synthesis and characterization of Schiff’s bases of sulfamethoxazole. Org Medicin Chem Lett. 2014;4(1):1–4. 10.1186/2191-2858-4-1 PMC3938971 24576663

[37] Bhat I, Mishra SK, James J, et al Antimicrobial studies of synthesized azetidinone derivatives from sulfamethoxazole moiety. J Chem Pharm Res. 2011;3(3):114–118.

[38] Cairns D Essentials of pharmaceutical chemistry. 4th ed. UK: Pharmaceutical Press; 2012.

[39] Semlali A, Jacques E, Rouabhia M, et al Regulation of epithelial cell proliferation by bronchial fibroblasts obtained from mild asthmatic subjects. Allergy. 2010;65(11):1438–1445. 20456314 10.1111/j.1398-9995.2010.02376.x

[40] Semlali A, Chakir J, Goulet JP, et al Whole cigarette smoke promotes human gingival epithelial cell apoptosis and inhibits cell repair processes. Periodont Res. 2011;46(5):533–541. 10.1111/j.1600-0765.2011.01370.x 21517857

[41] Bourara H, Hadjout S, Benabdelghani Z, et al Miscibility and hydrogen bonding in blends of poly (4-vinylphenol)/poly (vinyl methyl ketone). Polymers (Basel). 2014;6(11):2752–2763.

[42] Krumpfer JW, Giebel E, Frank E, et al Poly (methyl vinyl ketone) as a potential carbon fiber precursor. Chem Mater. 2017;29(2):780–788.

[43] Qiu Z, Ikehara T, Nishi T Miscibility and crystallization in crystalline/crystalline blends of poly (butylene succinate)/poly (ethylene oxide). Polymer (Guildf). 2003;44(9):2799–2806.

[44] Fox TG Influence of diluent and of copolymer composition on the glass temperature of a polymer system. Bull Am Phys Soc. 1952;1:123.

[45] Gordon M, Taylor JS Ideal copolymers and the second‐order transitions of synthetic rubbers. I. Non‐crystalline copolymers J Chem Technol Biotechnol. 1952;2(9):493–500.

[46] Vogel C, Wessel E, Siesler HW FT-IR spectroscopic imaging of anisotropic poly (3-hydroxybutyrate)/poly (lactic acid) blends with polarized radiation. Macromolecules. 2008;41(9):2975–2977.

[47] Benabdelghani Z, Etxeberria A. Hydrogen Bonds in Blends of Poly (vinylphenol‐co‐methylmethacrylate)/Poly (vinylmethylketone). Macromol Symp. 2012; 321(1):170-174.

[48] Kuo SW, Chang FC Miscibility and hydrogen bonding in blends of poly (vinylphenol-co-methyl methacrylate) with poly (ethylene oxide). Macromolecules. 2001;34(12):4089–4097.

[49] Wu Y-S, Wu Y-C, Kuo S-W Thymine- and adenine-functionalized polystyrene form self-assembled structures through multiple complementary hydrogen bonds. Polymers (Basel). 2014;6(6):1827–1845.

[50] Zhu B, He Y, Yoshie N, et al Partial phase segregation in strongly hydrogen-bonded and miscible blends. Macromolecules. 2004;37(9):3257–3266.

[51] Zaini E, Sumirtapura YC, Halim A, et al Formation and characterization of sulfamethoxazole-trimethoprim cocrystal by milling process. J Appl Pharmaceut Sci. 2017;7(12):169–173.

[52] Erizal E, Cahyati SY, Nurono SS, et al Effect of milling on solid state transformation of sulfamethoxazole. Int J Pharmacol. 2008;4:140–144.

[53] Knipe JM, Peppas NA Multi-responsive hydrogels for drug delivery and tissue engineering applications. Regenerat Biomater. 2014;1(1):57–65. 10.1093/rb/rbu006 PMC4669007 26816625

[54] Grinsted RA, Clark L, Koenig JL Study of cyclic sorption-desorption into poly (methyl methacrylate) rods using NMR imaging. Macromolecules. 1992;25(4):1235–1241.

[55] Ganji F, Vasheghani FS, Vasheghani FE Theoretical description of hydrogel swelling: A review Iran. Polym J. 2010;19:375–398.

[56] Porter TL, Stewart R, Reed J, et al Models of hydrogel swelling with applications to hydration sensing. Sensors. 2007;7(9):1980–1991. 28903209 10.3390/s7091980 PMC3841858

[57] Kim SJ, Lee KJ, Kim SI Swelling behavior of polyelectrolyte complex hydrogels composed of chitosan and hyaluronic acid. J Appl Polym Sci. 2004;93(3):1097–1101.

[58] Kim B, La Flamme K, Peppas NA Dynamic swelling behavior of pH‐sensitive anionic hydrogels used for protein delivery. J Appl Polym Sci. 2003;89(6):1606–1613.

[59] Peppas NA, Franson NM The swelling interface number as a criterion for prediction of diffusional solute release mechanisms in swellable polymers. PolymSci Part B Polym Phys. 1983;21(6):983–997.

[60] Comyn J. Introduction to polymer permeability and the mathematics of diffusion. In Comyn J. editor. Polymer permeability. Dordrecht: Springer; 1985. p. 1-10.

[61] Petty RD, Sutherland LA, Hunter EM, et al Comparison of MTT and ATP‐based assays for the measurement of viable cell number. Luminescence. 1995;10(1):29–34. 10.1002/bio.1170100105 7762413

[62] Petri W Sulfonamides, trimethoprim-sulfamethoxazole, quinolones, and agents for urinary tract infections In: Goodmans and Gilman’s the pharmacological basis of therapeutics. 12th ed. New York: The MaGraw-Hill Companies Inc; 2011 p. 1463–1476.

[63] Göktürk S, Çalışkan E, Talman RY, et al A study on solubilization of poorly soluble drugs by cyclodextrins and micelles: complexation and binding characteristics of sulfamethoxazole and trimethoprim. Sci World J. 2012;2012:1–12. 10.1100/2012/718791 PMC3353312 22649316

[64] Özdemir N, Erkin J Enhancement of dissolution rate and bioavailability of sulfamethoxazole by complexation with β-cyclodextrin. Drug Develop Ind Pharm. 2012;38(3):331–340. 10.3109/03639045.2011.604327 22059382

[65] Sekiguchi K, Obi N Studies on absorption of solid dispersions. I. A comparison of the behaviour of solid dispersions of sulphathiazole and that of ordinary sulphathiazole in man. Chem Pharm Bull. 1961;9:866–872.

[66] Premchandani TA, Barik BB Preparation and statistical optimization of alginate based stomach specific floating microcapsules of simvastatin. Acta Poloniae Pharm–Drug Res. 2012;69:751–761. 22876619

[67] Pandya VM, Patel JK, Patel DJ Effect of different stabilizer on the formulation of simvastatin nanosuspension prepared by nanoprecipitation technique. Res J Pharm Biol Chem Sci. 2010;1:910–917.

[68] Vyas A, Saraf S, Saraf S Encapsulation of cyclodextrin complexed simvastatin in chitosan nanocarriers: A novel technique for oral delivery. J Inclus Phenom Macrocycl Chem. 2010;66(3–4):251–259.

[69] Lin M, Wang H, Meng S, et al Structure and release behavior of PMMA/silica composite drug delivery system. J Pharmaceut Sci. 2007;96(6):1518–1526. 10.1002/jps.20809 17094137

[70] Masaro L, Zhu X Physical models of diffusion for polymer solutions, gels and solids. Prog Polym Sci. 1999;24(5):731–775.

[71] Reinhard CS, Radomsky ML, Saltzman WM, et al Polymeric controlled release of dexamethasone in normal rat brain. J Control Release. 1991;16(3):331–339.

[72] Cypes SH, Saltzman WM, Giannelis EP Organosilicate-polymer drug delivery systems: controlled release and enhanced mechanical properties. J Control Release. 2003;90(2):163–169. 12810299 10.1016/s0168-3659(03)00133-0

[73] Lee YY, Erdogan A, Rao SS How to assess regional and whole gut transit time with wireless motility capsule. J Neurogastroenterol Motil. 2014;20(2):265. 24840380 10.5056/jnm.2014.20.2.265 PMC4015195

[74] Degen L, Phillips S Variability of gastrointestinal transit in healthy women and men. Gut. 1996;39(2):299–305. 8977347 10.1136/gut.39.2.299 PMC1383315

[75] Schneider T, Keiblinger KM, Schmid E, et al Who is who in litter decomposition? Metaproteomics reveals major microbial players and their biogeochemical functions. ISME J. 2012;6(9):1749–1762. 22402400 10.1038/ismej.2012.11 PMC3498922

